# Effect of Cation−π Interactions on the Phase Behavior and Viscoelastic Properties of Polyelectrolyte Complexes

**DOI:** 10.1021/acs.macromol.4c02626

**Published:** 2025-04-16

**Authors:** Conner H. Chee, Aijie Han, Gileanna Ortiz, Lexi R. Knight, Jennifer E. Laaser

**Affiliations:** Department of Chemistry, 6614 University of Pittsburgh, 219 Parkman Avenue, Pittsburgh, Pennsylvania 15260, United States

## Abstract

Aromatic rings are a common feature of biological and synthetic polymers that form polyelectrolyte complexes and coacervates. These functional groups can engage in cation−π interactions; however, the impact of such interactions on the physical properties of polyelectrolyte complex materials is not well understood. Here, we investigate the effect of cation−π interactions on the phase behavior and viscoelasticity of polyelectrolyte complexes of poly­(styrenesulfonate) (PSS) and poly­(diallyldimethylammonium), which contain aromatic functional groups on every repeat unit of the PSS polyanion. We prepare samples with matched polymer and/or salt concentrations using salts with different cation−π interaction strengths. Characterization by turbidity, thermogravimetric analysis, and rheology reveals that salts that engage in stronger cation−π interactions destabilize coacervation and speed up the viscoelastic relaxation of the materials. By contrast, removing the aromatic ring by replacing PSS with poly­(2-acrylamido-2-methylpropanesulfonate removes the sensitivity of the phase behavior and viscoelasticity of the complexes to the cation−π interaction strength of the salt. These results reveal that cation−π interactions play a significant role in determining the phase behavior and viscoelasticity of polyelectrolyte complexes and coacervates made from polymers with aromatic functional groups and suggest that cation−π interactions may be a useful molecular handle for tuning coacervate properties.

## Graphical abstract

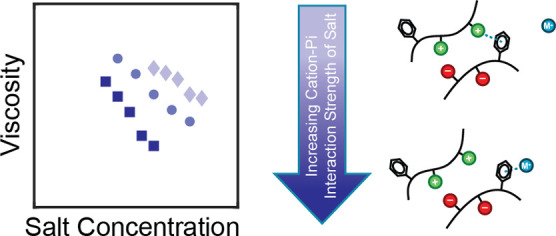

## Introduction

Oppositely charged polymers, when mixed in aqueous solution, can undergo associative phase separation to form materials called polyelectrolyte complexes or coacervates (PECs).^1,2^ These materials have highly tunable properties which have made them attractive for applications ranging from underwater adhesives^3,4^ to drug delivery^5–8^ and self-healing materials,^9^ as well as as models for biological condensates.^10,11^ Across these applications, both the phase behavior and viscoelasticity of the materials are critical for achieving their desired functions. To date, most studies have investigated the role of solution conditions, such as temperature,^12,13^ pH,^14–16^ and ionic strength.^14,17,18^ For many applications, however, such as those in physiological environments, these solution conditions are fixed and cannot be used as a handle to control the material properties. Understanding how to control the phase behavior and viscoelasticity of polyelectrolyte complexes and coacervates independent of the solution conditions is thus critical for functional materials design.

Recently, polymer design has emerged as a powerful way to control the phase behavior and viscoelasticity of PECs under fixed solution conditions. Advances in polymer synthesis have enabled preparation of polyelectrolyte complexes made from polymers with well-defined and systematically varied molecular weights,^19–21^ backbone chemistries,^14,22^ side chain functionalities,^23–26^ and sequences.^27,28^ These experiments have revealed that increasing molecular weight, charge density, and hydrophobicity all stabilize the complexes and favor phase separation. A number of studies have also investigated how these parameters affect the viscoelasticity of the materials. Decreasing the charge density of the polyelectrolytes, for example, has been found to speed relaxation and yield more liquid-like materials,^26,29^ while increasing the hydrophobic content slows relaxation and yields more solid-like materials.^26^ Together, these studies have highlighted the importance of molecular-scale chemical features of the polymer chains in determining the phase behavior and physical properties of polyelectrolyte complex materials.

One chemical feature that has received comparatively little attention in work on polyelectrolyte complexes to date is the presence of aromatic rings. Many of the polyelectrolytes used in fundamental studies of polyelectrolyte complexation, including poly­(styrenesulfonate)/poly­(diallyl­dimethyl­ammonium) (PSS/PDADMA), contain aromatic functional groups on a substantial fraction of the repeat units.^30–34^ Aromatic functional groups are also found in many of the intrinsically disordered proteins and other polypeptides that are known to undergo coacervation or liquid–liquid phase separation in biological environments.^4,35–37^ In these systems, there is emerging evidence that aromatic functional groups play a critical role in the complexation and phase separation process by engaging in cation-π interactions with positively charged functional groups elsewhere on the molecules.^35,36,38^ Cation-π interactions occur when cationic functional groups interact with the electron-rich face of an aromatic ring,^39^ and are known to impact biological processes as diverse as protein folding and molecular recognition.^40–42^ In synthetic polyelectrolytes that contain aromatic functional groups on many or all of the repeat units, favorable cation-π interactions between chains might similarly be expected to stabilize complex formation and slow viscoelastic relaxation. Testing this hypothesis, however, has been challenging, because it is difficult to control the aromatic content of the polymers without also affecting their hydrophobicity, backbone polarity, and/or secondary structure formation.^43^

An alternative method for probing the role of cation-π interactions is to investigate the behavior of the complexes in the presence of salts with different cation-π interaction strengths.^44^ In the gas phase, the cation-π interaction strength of cations of Group I metals increases with increasing cation charge density (K^+^ < Na^+^ < Li^+^).^39^ In solution, however, this trend reverses because the high charge density cations more tightly bind their hydration shells.^45–47^ This dependence has been exploited to show that cation-π interactions can play an important role in adhesion, with the adhesion between poly­(tryptophan) and poly­(lysine)-functionalized surfaces shown to decrease in the presence of potassium salts that form potassium-tryptophan interactions and out-compete the tryptophan-lysine interactions between the chains.^44^ In polyelectrolyte complexes, we similarly expect that if cation-π interactions stabilize PECs with aromatic functional groups, then the complexes should be destabilized in the presence of salts that form stronger cation-π interactions. Challenging PECs with salts with different cation-π interaction strengths should thus be a powerful tool for investigating the role of these interactions in polyelectrolyte complex materials.

Here, we use this approach to investigate the impact of cation-π interactions on the phase behavior and viscoelasticity of complexes of PSS and PDADMA, which contain aromatic functional groups on every side chain of the polyanion. For comparison, we also investigate the phase behavior and viscoelasticity of complexes of poly­(2-acrylamido-2-methylpropanesulfonate) (PAMPS) and PDADMA, which contain no aromatic functional groups. We first investigate the phase behavior of both polymer pairs in the presence of lithium, sodium, and potassium salts using optical turbidity and thermogravimetric analysis. We find that the stability of the PSS/PDADMA system does indeed depend on the cation-π interaction strength of the salt, with the stability increasing from K^+^ to Na^+^ to Li^+^. The stability of the PAMPS/PDADMA system, on the other hand, is independent of the type of salt. We then characterize the viscoelasticity of samples prepared above the binodal, where samples can be prepared at comparable salt and polymer concentrations despite changes in the position of the phase boundary.^33^ As in the phase behavior, the viscosities and relaxation times of the PSS/PDADMA system depend strongly on salt identity while those of the PAMPS/PDADMA system do not. These results show that cation-π interactions play an important role in both the phase behavior and physical properties of the PSS/PDADMA system, and should be taken into account in polyelectrolyte complexation experiments on polymers containing aromatic rings. They also suggest that aromatic content will be a useful molecular handle to tune the materials properties of PECs for functional applications, as described in more detail, below.

## Experimental Section

### Materials

Poly­(sodium 4-styrenesulfonate) (PSSNa, *M*_w_ = ∼200,000 g/mol, 20 wt %) solution, poly­(diallyldimethylammonium chloride) (PDADMAC, *M*_w_ = 200,000–350,000 g/mol, 23 wt %) solution, lithium bromide (LiBr), sodium bromide (NaBr), and potassium bromide (KBr) were purchased from Sigma-Aldrich. Poly­(2-acrylamido-2-methyl-1-propanesulfonic acid) (PAMPS, *M*_w_ = 800,000 g/mol, 10 wt %) solution was purchased from Fisher Scientific. PAMPS was neutralized with a 3 M NaOH solution until the pH was ∼7. PDADMAC, PSSNa, and neutralized PAMPS were dialyzed using standard RC dialysis tubing with a molecular weight cutoff of 6–8 kg/mol (Spectra/Por, 08–670D). To prevent water uptake, LiBr was stored in a glovebox until use. All other chemicals were used as received. All samples were prepared using Milli-Q water obtained from a Synergy UV water purification system from Millipore Sigma.

### Sample Preparation

Samples were prepared by direct mixing of dried PEC, salt or salt solution, and water. Bulk PECs were first prepared by mixing each polyelectrolyte pair above its critical salt concentration and precipitating the mixture from a low salt concentration solution.^48^ For the PSS/PDADMA PEC, PSSNa and PDADMAC were individually dissolved in 2.5 M KBr at a polymer concentration of 1 M (repeat unit basis). Stoichiometric amounts of the PSSNa and PDADMAC stock solutions were combined and stirred overnight to ensure complete mixing. This solution was then added dropwise to a 0.1 M KBr solution, resulting in precipitation of PSS/PDADMA complexes. The supernatant was decanted from the precipitated complexes and replaced with Milli-Q water, after which the complexes were allowed to soak for 12 h to remove excess salt. This process was repeated at least 5 times to ensure the complete removal of salt. The PEC was finally dried using a lyophilizer, yielding a white solid. Preparation of the PAMPS/PDADMA PECs followed the same procedure, except with an initial salt concentration during polyelectrolyte mixing of 1.4 M KBr. The final PAMPS/PDADMA PEC was a pale orange solid and was determined to have a density of 1.27 g/cm^3^ by density matching in hexane/chloroform mixtures.^49^ The dried PSS/PDADMA and PAMPS/PDADMA complexes were both verified to be stoichiometric by ^1^H NMR (see Supporting Information).

Rheology and TGA samples were then prepared by direct mixing of dried PEC, water, and salt (KBr, NaBr) or salt solution (LiBr). A summary of all targeted sample compositions is provided in the Supporting Information. For each sample, the requisite amounts of dried PEC, salt or salt solution, and water were combined in an Eppendorf tube and were vortexed between each addition. Samples were then centrifuged and inverted three times to ensure complete mixing. The samples were then allowed to equilibrate for at least 1 week before characterization. Rheology samples were periodically inverted during this equilibration period to ensure complete mixing.

### Optical Turbidity

The salt resistances of both PSS/PDADMA and PAMPS/PDADMA in the presence of different salts were measured by optical turbidity. Stock solutions of each polyelectrolyte were first prepared in Milli-Q water at a concentration of 50 mM (charged monomer basis). For experiments on PSS/PDADMA, salt stock solutions were prepared at concentrations of 4 M (NaBr and KBr) or 3.75 M (LiBr). For experiments on PAMPS/PDADMA, salt stock solutions were prepared at a concentration of 1.25 M for all salts. Samples were then prepared using an Opentrons OT-2 Pipetting Robot in a 96-well plate. The requisite amounts of polyanion stock solution, Milli-Q water, salt stock solution, and polycation stock solution were pipetted into each well, and were mixed by pipetting between each addition. After the final addition of the polycation, samples were manually pipetted several times to ensure complete mixing of phase-separated samples that clogged the robot’s pipet tip. All samples were prepared at a final total charged monomer concentration of 5 mM and a total volume of 200 μL. Optical turbidity measurements were immediately performed using an Infinite M1000Pro plate reader (Tecan) to measure the absorbance of each well at 500 nm.

### Thermogravimetric Analysis

Thermogravimetric analysis (TGA) measurements were carried out on a Q5000 IR Thermogravimetric Analyzer (TA Instruments) using a protocol adapted from Li et al.^17^ For each measurement, approximately 15 mg of sample was loaded onto a platinum pan. Samples prepared with NaBr and KBr were held at 25 °C for 5 min, heated to 130 °C at 20 °C/min and held at this temperature for 1 h to evaporate water. We note that this hold temperature is somewhat higher than used in previous work, but was found to be necessary to completely remove water from the PAMPS/PDADMA samples. The temperature was then ramped to 600 °C at a rate of 10 °C/min and the temperature was held for an additional 1 h to ensure complete removal of organic components of the sample. The temperature was then finally ramped to 680 °C at 10 °C/min to complete the measurement. For samples prepared with LiBr, continuing mass loss above 600 °C suggested that the salt was not stable at this temperature under the TGA conditions, so the protocol was modified to reduce the hold temperature for removal of the organic component to 500 °C and the final temperature was limited to 550 °C. All measurements were carried out under air to ensure the complete removal of the organic components. Measurements on standard solutions with known compositions indicated that this protocol yielded compositions accurate to within 1 wt %.

### Small-Amplitude Oscillatory Shear Rheology

Small-amplitude oscillatory shear rheology measurements were performed on an Anton Paar MCR-301 stress-controlled rheometer using a 25 mm sand-blasted parallel plate geometry. The parallel plate geometry was chosen to simplify loading of samples with a wide range of moduli while allowing use of a low sample volume. In each measurement, the frequency was swept from 600 to 0.1 rad/s, while the strain was increased simultaneously from 0.1 to 100%. The strain sweep protocol helps achieve adequate torques across the entire frequency range, and is known to improve sensitivity at low frequencies.^50^ Amplitude sweeps from approximately 0.1 to 100% strain at 10 rad/s were used to confirm that the entire frequency sweep was in the linear viscoelastic regime (see Supporting Information). Flow curves used to determine viscosity were run at shear rates that ranged from 0.1 to 500 s^–1^. To minimize effects from solvent evaporation, all measurements were carried out using an evaporation blocker, and the total measurement time was limited to approximately 1 h.

## Results

### Phase Behavior

The phase behaviors of both the PSS/PDADMA and PAMPS/PDADMA systems were quantified via optical turbidity, as shown in Figure 1. In turbidity experiments, high absorbance values indicate the formation of droplets or particles that have phase separated from solution and scatter light, while low absorbance values indicate the absence of phase separation and the formation of a homogeneous solution. As seen in Figure 1, the measured absorbance for both PSS/PDADMA and PAMPS/PDADMA samples was high at low salt concentrations and decreased at high salt concentrations. The transition point at which the absorbance first reaches its flat baseline at high salt concentrations indicates the salt resistance, or the salt concentration at which phase separated complexes are no longer formed. Importantly, there were significant differences in the point at which this transition occurred in the PSS/PDADMA and PAMPS/PDADMA systems, and its dependence on the identity of the salt used to set the ionic strength. First, the salt resistance for the PSS/PDADMA system was higher than that of the PAMPS/PDADMA system regardless of the salt identity. This indicates that the PSS/PDADMA system forms more stable complexes than the PAMPS/PDADMA system. Second, the salt resistance of the PAMPS/PDADMA system was effectively independent of salt identity, with all three salts giving salt resistances of approximately 0.75–0.8 M. The salt resistance of the PSS/PDADMA system, on the other hand, varied significantly with the salt identity, with the salt resistance increasing from 1.8 M for samples made with KBr to 2.4 M for samples made with LiBr.

**Figure 1:**
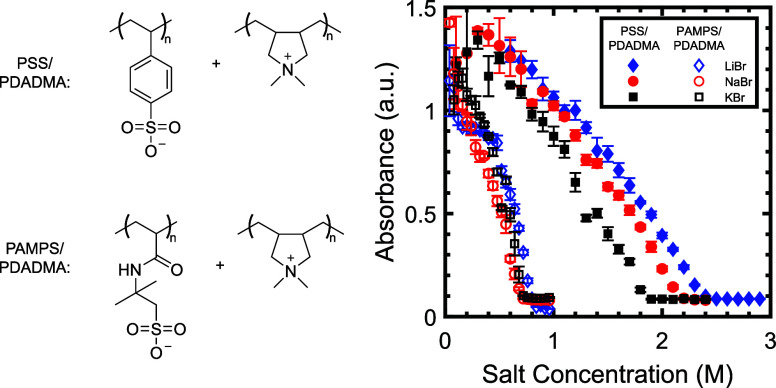
Optical turbidities of PSS/PDADMA and PAMPS/PDADMA samples prepared at a total monomer concentration of 0.005 M. Error bars indicate the standard deviation of measurements on samples prepared in triplicate.

The phase behavior was further characterized by thermogravimetric analysis (TGA). TGA was used to determine the mass fractions of water, polymer, and salt in both the coacervate and supernatant phases of several samples for each polymer and salt combination, as described in the Supporting Information. The mass fractions measured by TGA were then converted to volume fractions using the bulk densities of each component (1.27 g/cm^3^ for both PSS/PDADMA^49^ and PAMPS/PDADMA complexes, 1.00 g/cm^3^ for water, 3.46 g/cm^3^ for LiBr, 3.21 g/cm^3^ for NaBr, and 2.75 g/cm^3^ for KBr). The resulting phase diagrams are shown in Figure 2a,b, respectively. In these phase diagrams, filled symbols indicate the composition of the polymer-rich coacervate phase, while open symbols indicate the composition of the polymer-poor supernatant. These points form the concave-down binodal curve typically observed in polyelectroyte complex systems.^30,51^ The tie lines connecting the compositions of the coacervate and supernatant phases for PSS/PDADMA samples prepared with NaBr and KBr are relatively flat, consistent with prior reports of athermal mixing in this system.^52^ The tie lines for PSS/PDADMA samples prepared with LiBr and PAMPS/PDADMA samples prepared with all three salts, on the other hand, have slightly positive slopes, indicating a slight preference for salt to partition into the coacervate phase. The critical point, or the top point on the binodal curve, of both polymer pairs shifts to lower volume fractions of salt as the salt is changed from KBr to LiBr. For direct comparison to turbidity measurements, however, the phase diagram must be plotted in terms of the molar concentrations of the components, as shown in Figure 2c,d. In this representation, the binodal curve for the PSS/PDADMA system shifts upward as the salt is changed from KBr to LiBr, while that of the PAMPS/PDADMA system is insensitive to the identity of the salt. Both turbidity and TGA thus show that PSS/PDADMA complexes are destabilized by salts with higher cation-π interaction strengths while PAMPS/PDADMA complexes are not, suggesting that cation-π interactions play an important role in the phase behavior of the PSS/PDADMA system.

**Figure 2:**
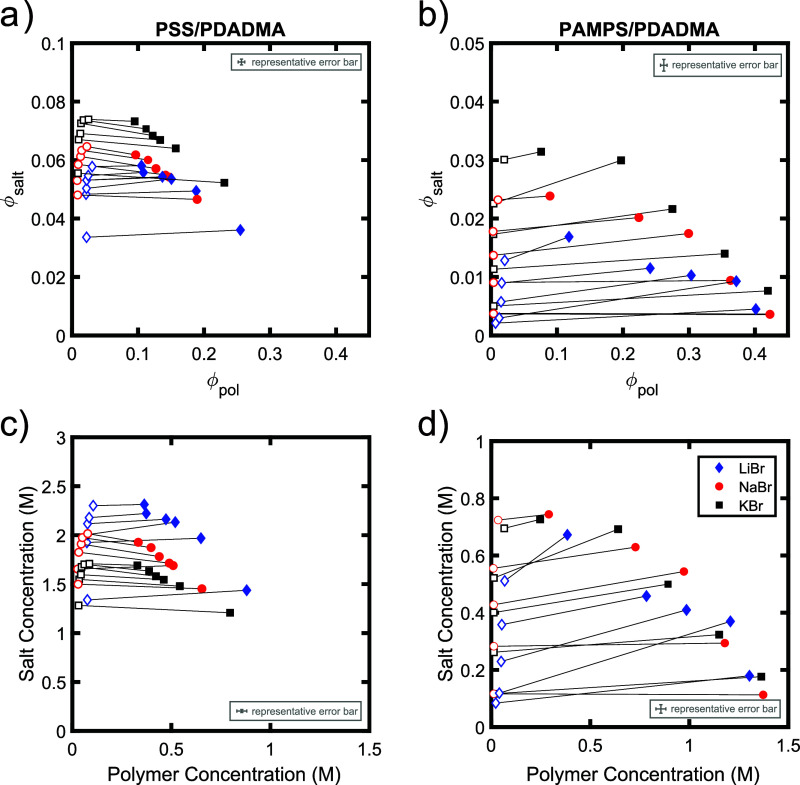
Phase diagrams of a (a, c) PSS/PDADMA and (b,d) PAMPS/PDADMA samples prepared with LiBr (blue diamonds), NaBr (red circles) and KBr (black squares), plotted as a function of (a, b) the volume fractions and (c, d) the molar concentrations of polymer and salt. Representative error bars indicate estimated uncertainties in the composition from repeated TGA measurements on a subset of the samples (see Supporting Information).

### Viscoelasticity

To determine whether cation-π interactions also impact the viscoelasticity of polyelectrolyte complexes and coacervates, the viscosities and dynamic moduli of PSS/PDADMA and PAMPS/PDADMA samples prepared with all three salts were characterized by small-amplitude oscillatory shear rheology. Two series of samples were prepared for each polymer and salt pair. In the first series of samples, the polymer concentration was held constant while the salt concentration was varied. In the second series of samples, the salt concentration was held constant while the polymer concentration was varied. Importantly, all samples were prepared in the single-phase regime of the phase diagram (above the binodal) to ensure that the polymer and salt concentrations of the samples could be controlled independently and that phase separation did not impact the measured viscoelasticities. Plots of all prepared sample concentrations, and where they fall relative to the binodal, are presented in the Supporting Information.

Representative flow curves of PSS/PDADMA and PAMPS/PDADMA samples prepared at a volume fraction of polymer of 0.15 and salt concentrations of 2.125 M (for PSS/PDADMA samples) and 1.000 M (for PAMPS/PDADMA samples) are shown in Figure 3. At low shear rates, below 1 s^–1^, the viscosity of each sample is constant. At higher shear rates, above 1–10 s^–1^, the viscosity decreases slightly. This shear thinning behavior arises from disruption of intermolecular interactions between the polyelectrolytes at high shear rates. While the shapes of the flow curves for PSS/PDADMA and PAMPS/PDADMA are very similar, however, the magnitudes of the viscosities and their dependence on salt identity are very different. The viscosity of the PSS/PDADMA sample prepared with KBr is an order of magnitude smaller than that of the sample prepared with LiBr. In contrast, the viscosities of the PAMPS/PDADMA samples decrease by less than a factor of 2 across the same series of salts. Similar trends were observed for samples prepared at other salt and polymer concentrations (see Supporting Information). This result is consistent with the picture that salts that engage in more favorable cation-π interactions can disrupt interactions between the chains when the polyelectrolytes contain aromatic functional groups.

**Figure 3:**
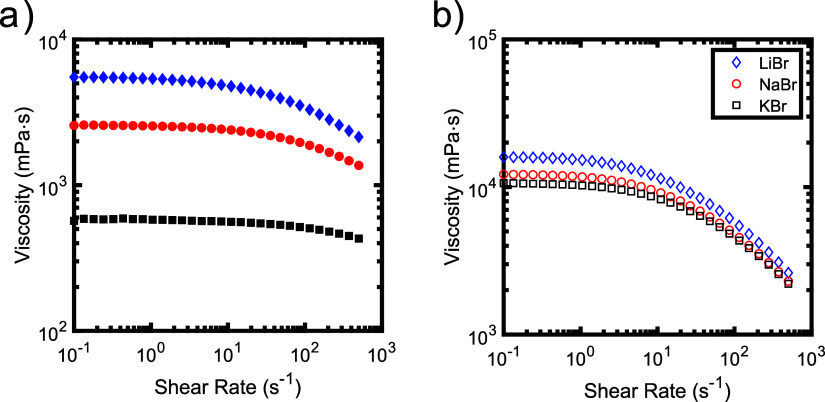
Flow curves of samples composed of (a) PSS/PDADMA and (b) PAMPS/PDADMA prepared at salt concentrations of 2.125 and 1.00 M, respectively. All samples were prepared at a volume fraction of polymer of 0.15.

The zero-shear viscosity of each sample was determined from the average of the viscosities measured at shear rates between ∼0.10–1.05 s^–1^. The zero shear viscosities for the series of samples prepared with constant polymer concentration (ϕ_pol_ = 0.15) and varying salt concentrations are plotted in Figure 4. As seen in this figure, the zero shear viscosity decreases with increasing salt concentration for all samples. Additionally, the differences in dependence on salt identity for the PSS/PDADMA and PAMPS/PDADMA samples reported in Figure 3 persist across all salt concentrations studied. Cation-π interactions thus appear to impact the coacervate viscosity over a wide range of solution conditions.

**Figure 4:**
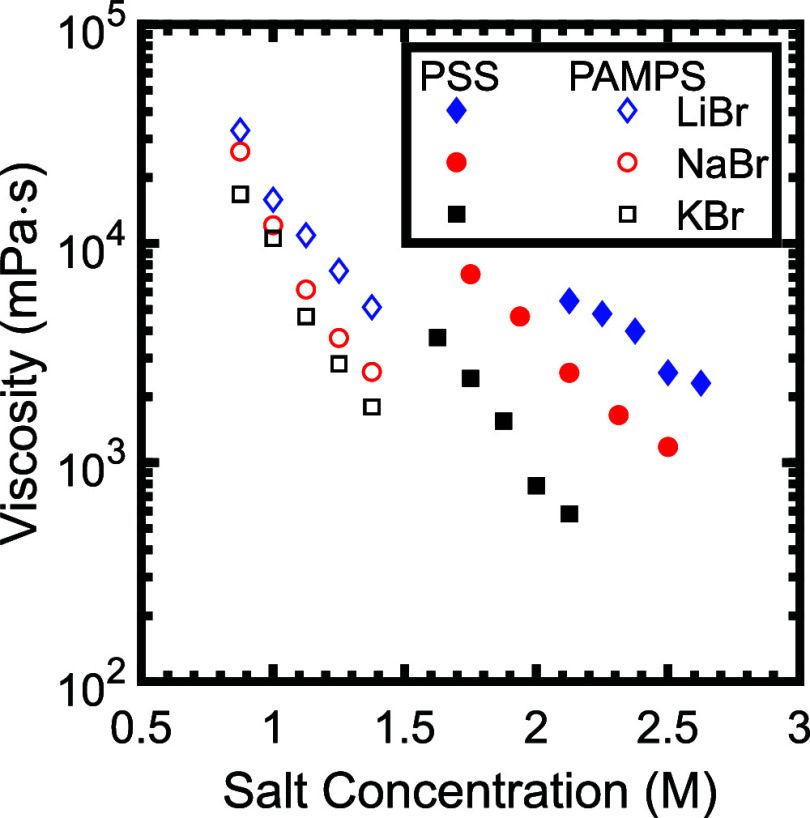
Zero shear viscosities of PSS/PDADMA (filled symbols) and PAMPS/PDADMA (open symbols) samples prepared at constant polymer concentration (ϕ_pol_ = 0.15) and varying salt concentration. Error bars are smaller than the symbol size (see Supporting Information).

Changes in viscosity can result either from changes in terminal relaxation time or from changes in modulus. To determine which of these factors is responsible for the changes in viscosity reported in Figure 4, frequency sweeps were additionally conducted on all samples. Representative frequency sweeps for PSS/PDADMA and PAMPS/PDADMA samples prepared with NaBr are shown in Figure 5. At low frequencies, all samples exhibited the *G*′∼ ω^2^ and *G*″ ∼ ω scaling characteristic of viscoelastic liquids in the terminal flow regime. Additionally, within each polymer pair, increasing the salt concentration decreased the dynamic moduli at each frequency. These data were analyzed via time-salt superposition to extract the shift factors and characteristic relaxation times and moduli of each sample. To enable direct comparison between samples with different salts, the data from the highest salt concentration sample for each polymer/salt pair was first shifted onto a Maxwell model with τ = 1 s and *G* = 1 Pa. This data was then used as the reference trace for all remaining salt concentrations. This procedure effectively shifts all frequency sweeps to a common reference, and yields horizontal shift factors that approximate the terminal relaxation times of the samples and vertical shift factors that approximate the inverse of the expected modulus at the crossover point. We note that the frequencies and moduli at the crossover points were also estimated from the intersection of power law fits to the low-frequency portions of *G*′ and *G*″; while we focus here on the superposition analysis, this fitting approach yielded very similar results (see Supporting Information).

**Figure 5:**
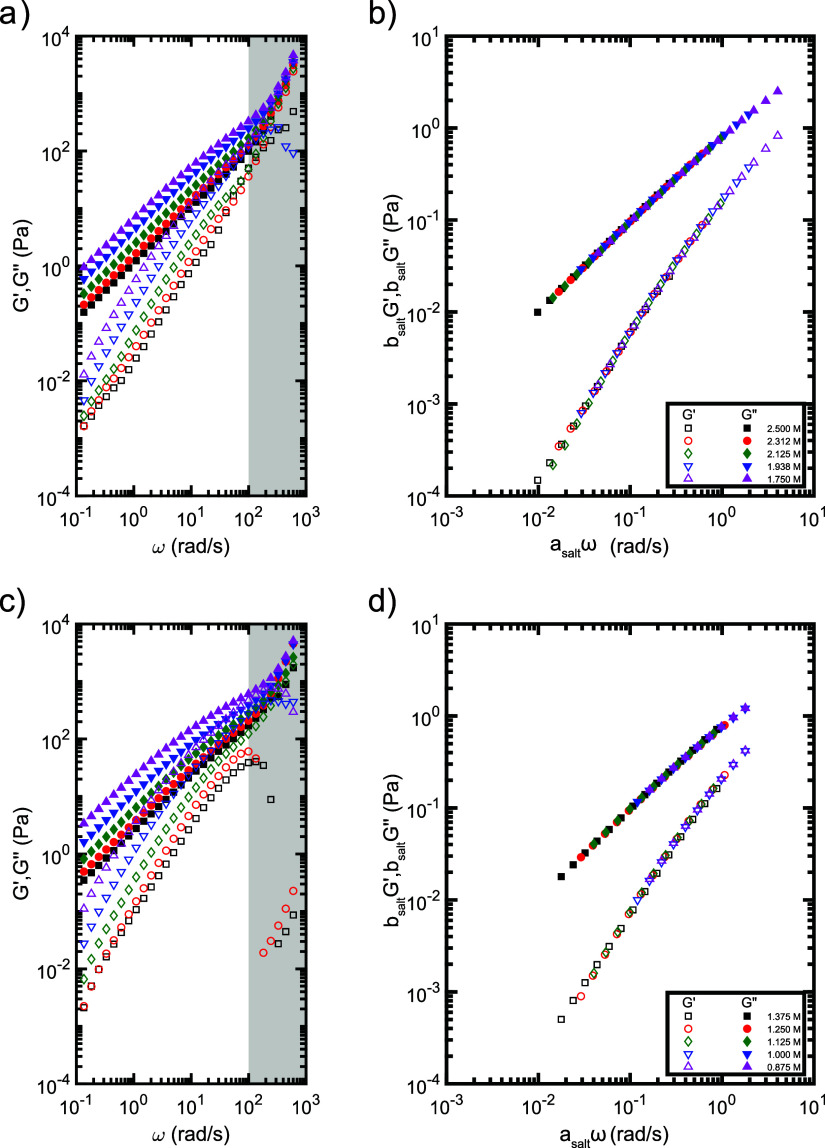
(a, c) Frequency sweeps and (b, d) master curves of (a, b) PSS/PDADMA and (c, d) PAMPS/PDADMA samples prepared with NaBr. Data in the shaded region was impacted by the inertial limit of the rheometer and was excluded from the superposition analysis. Analogous plots for samples prepared with LiBr and KBr are reported in the Supporting Information.

The master curves resulting from the superposition analysis are shown in Figure 5b,d, and the horizontal and vertical shift factors for all polymer/salt pairs are summarized in Figure 6. As seen in this figure, increasing the salt concentration led to a decrease in the horizontal shift factors for all polymer/salt pairs. The horizontal shift factors of the PSS/PDADMA system were strongly dependent on the salt identity, with samples prepared with KBr having horizontal shift factors (and, correspondingly, relaxation times), approximately an order of magnitude lower than those prepared with LiBr at the same salt concentration. By contrast, the horizontal shift factors of the PAMPS/PDADMA system were largely independent of the salt identity. These results are consistent with the trend observed in the viscosity data, and suggest that cation-π interactions play a role in the viscoelastic relaxation of polyelectrolyte complexes containing aromatic functional groups. Interestingly, we note that neither increasing the salt concentration nor changing the salt identity led to significant changes in the vertical shift factors. This result indicates that salt primarily affects the relaxation time of the materials, rather than their moduli.

**Figure 6:**
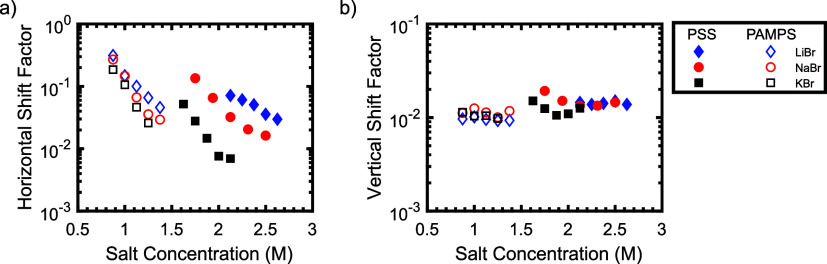
(a) Horizontal and (b) vertical shift factors for PSS/PDADMA and PAMPS/PDADMA samples prepared with varying salt concentrations at a polymer concentration of ϕ_pol_ = 0.15.

Finally, complementary experiments were carried out to investigate the impacts of varying the polymer concentration in the samples. The zero-shear viscosities for samples prepared with constant salt concentrations and varying polymer concentrations are plotted in Figure 7, and the shift factors obtained from small-amplitude oscillatory shear rheology are plotted in Figure 8. We note that re-entrant phase separation was observed in the PSS/PDADMA samples at high salt and polymer concentrations,^53^ which prevented preparation of homogeneous samples at the same salt concentrations for all salts. As such, PSS/PDADMA samples made with LiBr were prepared at a salt concentration of 2.4 M, those with NaBr were prepared at a salt concentration of 2.1 M, and those with KBr were prepared at a salt concentration of 1.875 M. Samples prepared with PAMPS/PDADMA were prepared at a salt concentration of 1.25 M for all salts. This experimental design makes direct comparison of the absolute viscosities and shift factors between different salts challenging, but still allows interpretation of the trends in these values with changes in polymer concentration.

**Figure 7:**
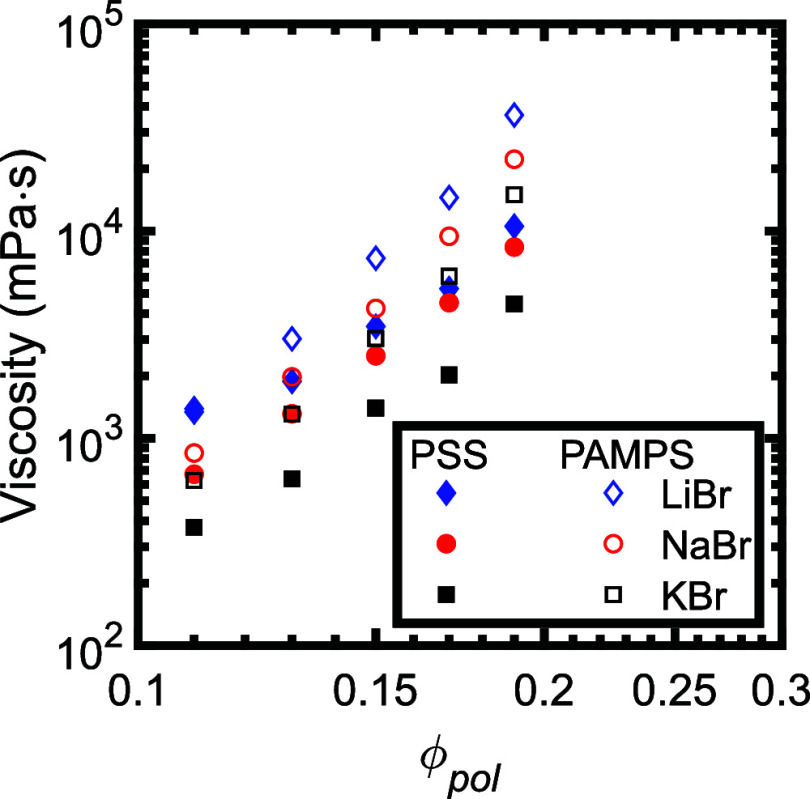
Zero shear viscosities of PSS/PDADMA (filled symbols) and PAMPS/PDADMA (open symbols) samples prepared at constant salt concentration and varying polymer concentrations. As noted in the text, PAMPS/PDADMA samples were prepared at a salt concentration of 1.25 M, while the salt concentration for PSS/PDADMA samples was varied from 2.4 M for LiBr to 2.1 M for NaBr to 1.875 M for KBr.

**Figure 8:**
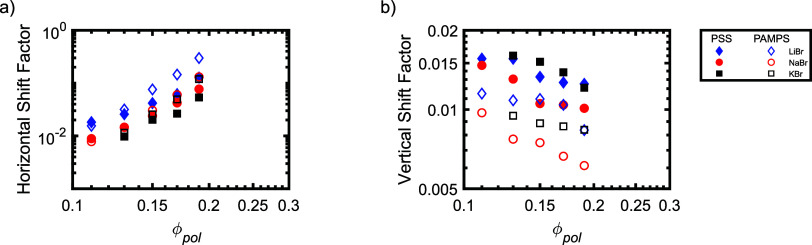
(a) Horizontal and (b) vertical shift factors for PSS/PDADMA (filled symbols) and PAMPS/PDADMA (open symbols) samples prepared at different polymer concentrations. As noted in the text, PAMPS/PDADMA samples were prepared at a salt concentration of 1.25 M, while the salt concentration for PSS/PDADMA samples was varied from 2.4 M for LiBr to 2.1 M for NaBr to 1.875 M for KBr.

As shown in Figure 7, the zero-shear viscosities of the samples increased by more than an order of magnitude with only a 2-fold increase in polymer concentration for all polymer/salt pairs. Analysis of the shift factors in Figure 8 shows that this strong dependence of the viscosity on the polymer concentration results primarily from increases in the terminal relaxation time (as reflected by the horizontal shift factor), with much smaller contributions from the changes in the modulus (as reflected by the inverse of the vertical shift factors). Quantitatively, the horizontal shift factors are found to scale as *a*_pol_ ∼ ϕ_pol_^3.5^ to ϕ_pol_^5.9^ while the vertical shift factors scale as *b*_pol_ ∼ ϕ_pol_^–0.3^ to ϕ_pol_^–0.8^ (see Supporting Information). The strong scaling of the horizontal shift factors (and thus relaxation times) with polymer concentration is consistent with previous reports on PSS/PDADMA coacervates prepared with KBr,^33^ and highlight the importance of associative interactions between the polymer chains in both the PSS/PDADMA and PAMPS/PDADMA systems, as discussed in more detail below.

## Discussion

Here, we investigated the role of cation-π interactions in the phase behavior and viscoelasticity of polyelectrolyte complexes by challenging the complexes with salts with different cation-π interaction strengths. Experiments were carried out with both polyelectrolytes containing aromatic side chains (PSS/PDADMA) and polyelectrolytes without aromatic side chains (PAMPS/PDADMA). We found that the salt resistances and viscosities of the PSS/PDADMA complexes decreased in the presence of salts with stronger cation-π interactions, and that this decrease in viscosity was driven by a decrease in the terminal relaxation time of the materials. By contrast, the salt resistances, viscosities, and relaxation times of the PAMPS/PDADMA samples exhibited little dependence on the cation-π interaction strength of the salt. Because the salt identity only affects the behavior of the PSS/PDADMA system, we can exclude other ion-specific effects, such as Hofmeister effects,^54^ which do not explicitly involve interactions between the cations and the aromatic rings. Instead, the fact that the cation-π interaction strength of the salt changes the phase behavior and viscoelasticity of samples containing aromatic rings but not of samples that do not contain aromatic rings suggests that cation-π interactions play an important role in polyelectrolyte complexation and relaxation when aromatic functional groups are present. We posit that when one polyelectrolyte in the mixture contains aromatic rings, favorable cation-π interactions between the chains stabilize the complexes and form associative interactions that slow relaxation. When salts that can engage in strong cation-π interactions are added to the system, they can out-compete these polymer–polymer interactions and destabilize the complexes and speed up their relaxation dynamics.

From a fundamental perspective, this is important because a significant amount of work on polyelectrolyte complexes and coacervates has been carried out using polyelectrolytes with aromatic functional groups. The PSS/PDADMA system has been used extensively for fundamental studies on both the phase behavior and viscoelasticity of these materials.^30,33,34,55,56^ PSS has also been used in complexation studies with a number of other polycations, including poly­(dimethylaminoethyl methacrylate),^57^ poly­(allylamine hydrochloride),^52,58^ and various poly­(vinylpyridines).^31^ Other studies have used polyelectrolytes containing aromatic functional groups primarily on the polycation, including both functionalized poly­(vinylpyridines)^31^ and polycations like poly­(vinyl benzyl trimethylammonium chloride).^59^ Across most of this work, the behavior of the complexes has been interpreted primarily in terms of electrostatic interactions, while contributions from cation-π interactions have largely been ignored. As our data reveals, however, cation-π interactions likely also play an important role in complexes of polyelectrolytes with aromatic functional groups, and their contributions should be considered when interpreting data from these systems. From an applied perspective, our data also suggests that cation-π interactions (and aromatic functional groups more generally) are potentially a useful design parameter for achieving targeted properties, especially in complex solution environments.

From an experimental design perspective, our approach offers a simple way to test for the role of cation-π interactions without modifications to the polymers. The phase behavior and physical properties of the complexes need only be assessed in the presence of two to three different salts with different cation-π interaction strengths. As long as all samples are prepared at the same salt and polymer concentration, the strengths of the polymer–polymer and polymer-salt electrostatic interactions should be comparable, and any differences observed can be attributed to contributions from cation-π interactions. This approach is significantly simpler than having to synthesize multiple polymers with and without aromatic functional groups, or in biological materials, preparing different aromatic and nonaromatic protein mutants. We do note that this approach cannot necessarily distinguish between contributions from cation-π interactions and other associative interactions between the aromatic groups on the polymers, such as π–π interactions. The salts must interact with the aromatic groups on the polymer through cation-π interactions, but they could destabilize the complexes by disrupting either cation-π or π–π interactions between the chains. We suspect that in the PSS/PDADMA system, the interchain interactions involving the aromatic groups are primarily cation-π interactions, because π–π interactions would require close association of like-charged units, which should be energetically unfavorable. However, further experiments on polymer systems in which the placement of the aromatic groups is varied (either by placing them on the polycation, or by placing them on separate repeat units from the charged sites) will be necessary to rigorously distinguish these two mechanisms.

While the primary focus of this work is on the importance of cation-π interactions, we note that investigating samples in the single-phase regime offers a unique opportunity to test models for coacervate viscoelasticity by enabling the polymer and salt concentrations of the materials to be varied independently.^33^ The rheology of polyelectrolyte complexes is typically described in terms of the “sticky Rouse” model, in which electrostatic stickers between chains slow their relaxation.^19,60^ We find that the viscosities of samples prepared at constant salt concentration scale with polymer concentration as approximately η ∼ ϕ_pol_^3.8–4.5^ for PSS/PDADMA and η ∼ ϕ_pol_^5.8–6.0^ for PAMPS/PDADMA (see Supporting Information), consistent with the sticky Rouse predictions of η ∼ ϕ_pol_^4.2^ and η ∼ ϕ_pol_^6.0^ for systems without and with bond renormalization, respectively.^61^ Bond renormalization may not be critical in the PSS/PDADMA system, because the additional binding sites provided by the aromatic rings make it easy for cations on PDADMA to find a new binding partner when a binding interaction is broken.

Interestingly, while the sticky Rouse model successfully reproduces the strong observed dependence of viscosity on ϕ_pol_,^33,61^ we note that its validity in describing coacervate systems - and particularly those with closely spaced electrostatic stickers, which should not associate strongly at high salt concentrations - has been called into question.^62^ It has alternatively been proposed that coacervates at high salt concentration should behave like quasi-neutral polyelectrolyte solutions;^63^ this picture, however, does not adequately explain the dependence on salt concentration in the single-phase regime or the strong scaling of relaxation time with polymer concentration. Our work thus reveals that salt-mediated sticky interactions must play a role in slowing chain relaxation. Whether these interactions occur specifically between oppositely charged ionic sites, however, is less clear. It has been suggested, for example, that the relaxation dynamics of complex coacervates can have significant contributions from hydrophobic interactions.^26,64^ At the compositions investigated in this work, the samples contain as few as 10 water molecules per ionic species, in which limit hydrophobic associations may play an important role and may be highly sensitive to the salt concentration in solution. Models of sticky relaxation dynamics that allow for sticky sites on every repeat unit and/or that have effectively one intermolecular sticky site per correlation blob may also be a useful alternative model for coacervate relaxation.^65^ Finally, we note that extremely strong scaling of diffusion coefficients with polymer concentration have also been observed in concentrated polyelectrolyte solutions with volume fractions of polymer above approximately 10%.^66^ The strong dependence of viscosity and relaxation time on polymer concentration observed in our work thus could also arise from a more universal relaxation behavior of polyelectrolytes. Thus, while our work makes it clear that salt-mediated sticky interactions do matter in relaxation of complex coacervates, further work will be needed to clarify the molecular-scale mechanisms at play.

Resolving many of these questions will require further experiments on well-defined synthetic polymer systems. While PSS and PAMPS are widely available polyanions, they have significantly different backbone and side chain structures, and thus different hydrophobicities. This makes interpretation of the absolute differences between the behaviors of PSS/PDADMA and PAMPS/PDADMA coacervates, such as the differences in salt resistance shown in Figure 1, difficult. Direct comparison of polymers synthesized with more similar backbones and side chains would provide additional insight into the relative roles of hydrophobic and aromatic interactions. The commercial polymers used in this work also typically have broad molecular weight distributions, and the average molecular weights of the PSS and PAMPS polymers differed by a factor of 4. The sample preparation method used here ensures that all samples of a given polymer pair have the same molecular weight distribution, so molecular weight effects cannot explain the different responses to the different salt identities. Direct comparison of the absolute viscoelasticities of different polymer systems, however, and testing of models for coacervate viscoelasticity, will require further experiments on polymer systems with narrow dispersities and comparable molecular weights. These experiments, among others, will help paint a fuller picture of the role of cation-π interactions in polyelectrolyte complexes and coacervates and how they are mediated by other features of the constituent polyelectrolytes.

## Conclusions

In this work, we have shown that cation-π interactions can play a significant role in the phase behavior and viscoelasticity of polyelectrolyte complexes and coacervates. We prepared PECs that either contained aromatic functional groups (PSS/PDADMA) or did not contain aromatic functional groups (PAMPS/PDADMA) and challenged them with salts with different cation-π interaction strengths (LiBr < NaBr < KBr). Increasing the cation-π interaction strength of the salt was found to destabilize coacervation, reduce viscosity, and speed relaxation of the materials containing aromatic functional groups but not of those that did not contain aromatic functional groups. The strategies used in this work, namely testing the role of cation-π interactions by changing the identity of the salt and preparing samples in the single-phase regime to decouple effects due to changes in composition, salt concentration, or salt identity from those due to changes in polymer concentration, offer a straightforward way to probe cation-π interactions without modifying the component polymers. Interestingly, experiments in the single-phase regime highlighted not only the impact of cation-π interactions, but also the strong dependence of the viscoelasticity of the materials on both the polymer concentration and the salt concentration. These results reinforce the idea that salt-mediated interactions matter for the viscoelasticity of these materials, but raise new questions about the molecular-scale mechanisms at play. Further experimental and theoretical work on both cation-π and other associative interactions will offer important opportunities to deepen fundamental insights about the chemical interactions that determine the physical properties of polyelectrolyte complex materials. This insight should in turn provide new design rules for making materials targeted for specific applications.

## Supplementary Material



## Data Availability

The raw data files for all samples are available via the authors’ institutional data repository, D-Scholarship@Pitt, at https://d-scholarship.pitt.edu/47028/.

## Notes

Supplemental characterization data (NMR, turbidity, TGA, and rheology) for all samples, and details of the superposition and scaling analyses used to analyze rheology data (PDF)
