# Supplementary material for: Effect of Cation−π Interactions on the Phase Behavior and Viscoelastic Properties of Polyelectrolyte Complexes
Source: Macromolecules. 2025 Apr 16;58(10):5177–86. doi: 10.1021/acs.macromol.4c02626 (PMC12120988; doi:10.1021/acs.macromol.4c02626)
Supplement: Supplementary file 1 [file ma4c02626_si_001.pdf]

Supporting Information: Effect of Cation-π Interactions on the Phase Behavior and Viscoelastic Properties of Polyelectrolyte Complexes

Conner H. Chee, Aijie Han, Gileanna Ortiz, Lexi R. Knight, and Jennifer E. Laaser*

Department of Chemistry, University of Pittsburgh, 219 Parkman Ave., Pittsburgh, PA 15260, United States

E-mail: j.laaser@pitt.edu

Supplemental Results

Nuclear Magnetic Resonance

The stoichiometries of the parent PSS/PDADMA and PAMPS/PDADMA PECs used to prepare the TGA and rheology samples were verified by ^{1}H-NMR. The dried PECs were dissolved in a solution of KBr in D_2O ([KBr] = 1.3 M for PAMPS/PDADMA and 2.5 M for PSS/PDADMA), and spectra were acquired on a Bruker Avance III 400 MHz spectrometer. Spectra of both PECs are shown in Figure S1.

The mole fraction of charged repeat units coming from PSS in the PSS/PDADMA PEC

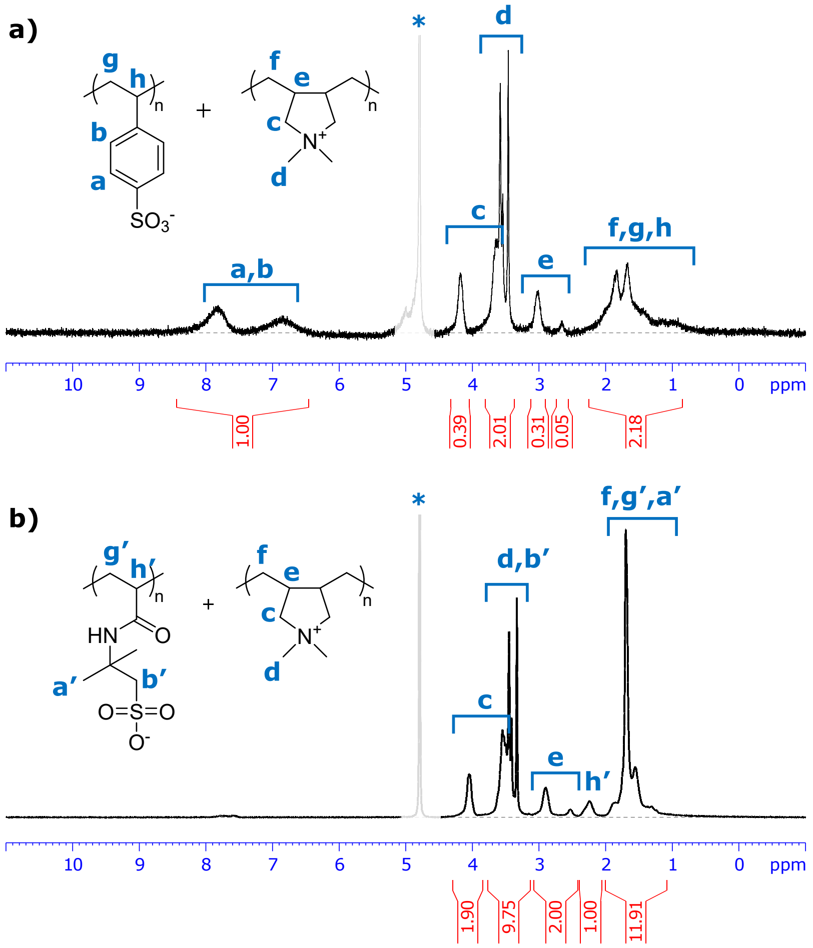

Figure S1: ^{1}H-NMR spectra (D_2O, 400 MHz) of the parent (a) PSS/PDADMA and (b) PAMPS/PDADMA PECs used in preparation of TGA and rheology samples.

was determined using

$$x_{PSS} = \frac{PSS}{PSS + PDADMA} = \frac{4I_{aromatic}}{4I_{aromatic} + (I_{aliphatic} - \frac{3}{4}I_{aromatic})} \quad (1)$$

where $I_{aromatic}$ is the total intensity in the aromatic region (6-8 ppm) and I_{aliph} is the total intensity in the aliphatic region (1-4.5 ppm).[S1,S2] Using this analysis, the PSS/PDADMA PEC was determined to contain 48.7% PSS (molar basis). Direct comparison of the integral in the aromatic region (corresponding to four protons on the PSS ring) to that at 4.2 ppm (corresponding to half of the signal from the four pyrollidinium methylene protons, c) yields x_{PSS} = 55%. The PSS/PDADMA PEC was thus stoichiometric to within the accuracy

measurable by NMR.

The mole fraction of charged repeat units coming from PAMPS in the PAMPS/PDADMA PEC was similarly determined using

$$x_{PAMPS} = \frac{I_{3.2-3.7} - 4I_{4.2}}{I_{3.2-3.7} - 3I_{4.2}} \tag{2}$$

where $I_{3.2-3.7}$ is the intensity of the peak between 3.2 and 3.7 ppm corresponding to the the 6 PDADMA methyl protons (d) , half the signal from the pyrollidinium methylene protons (c), and the PAMPS methylene protons adjacent to the sulfonate (b'), and $I_{4.2}$ is the intensity of the peak at 4.2 ppm corresponding to the other half of the signal from the pyrollidinium methylene protons (c).[S2] Using this analysis, the PAMPS/PDADMA PEC was determined to contain 53.1% PAMPS (molar basis). This stoichiometry was verified by comparing the intensities of the peaks between 2.1 and 2.4 ppm (PAMPS backbone methine, h') and 2.4-3 ppm (PDADMA backbone methine, e), which gave a composition of 50% PAMPS. The PAMPS/PDADMA PEC was thus also stoichiometric to within the accuracy measurable by NMR.

Thermogravimetric Analysis

Thermogravimetric analysis (TGA) traces for all samples used to generate the phase diagrams shown in the main text are presented in Figs. S2-S3. As noted in the main text, the TGA protocol included isotherms at 130 °C and 600 °C to drive off water and polymer, respectively. The mass loss between 25 and 140 °C was taken as the mass of water in the sample, the loss between 140 and 610 °C was taken as the mass of polymer in the sample, and the remaining mass at the end of the run was taken as the mass of salt. The initial mass loss observed in each trace at 25 °C was attributed to evaporation of water during the 5 min hold before the start of the temperature ramp. This mass loss was included in the mass fraction of water for each sample.

Repeat measurements were carried out on a subset of the TGA samples to assess the reproducibility in the determined compositions. Representative TGA traces illustrating the reproducibility of the TGA measurements are shown in Fig. S4. Across all repeated samples, the mass fractions of polymer and salt typically agreed to within 1 wt% (and often less than 0.1 wt%). This translated into estimated uncertainties in the volume fractions of PEC and salt of approximately ± 0.005 and ± 0.002, and uncertainties in the molar concentrations of polymer and salt of ± 0.025 M and ± 0.015 M, respectively. These values were used to construct the representative error bars shown on Fig. 2 in the main text.

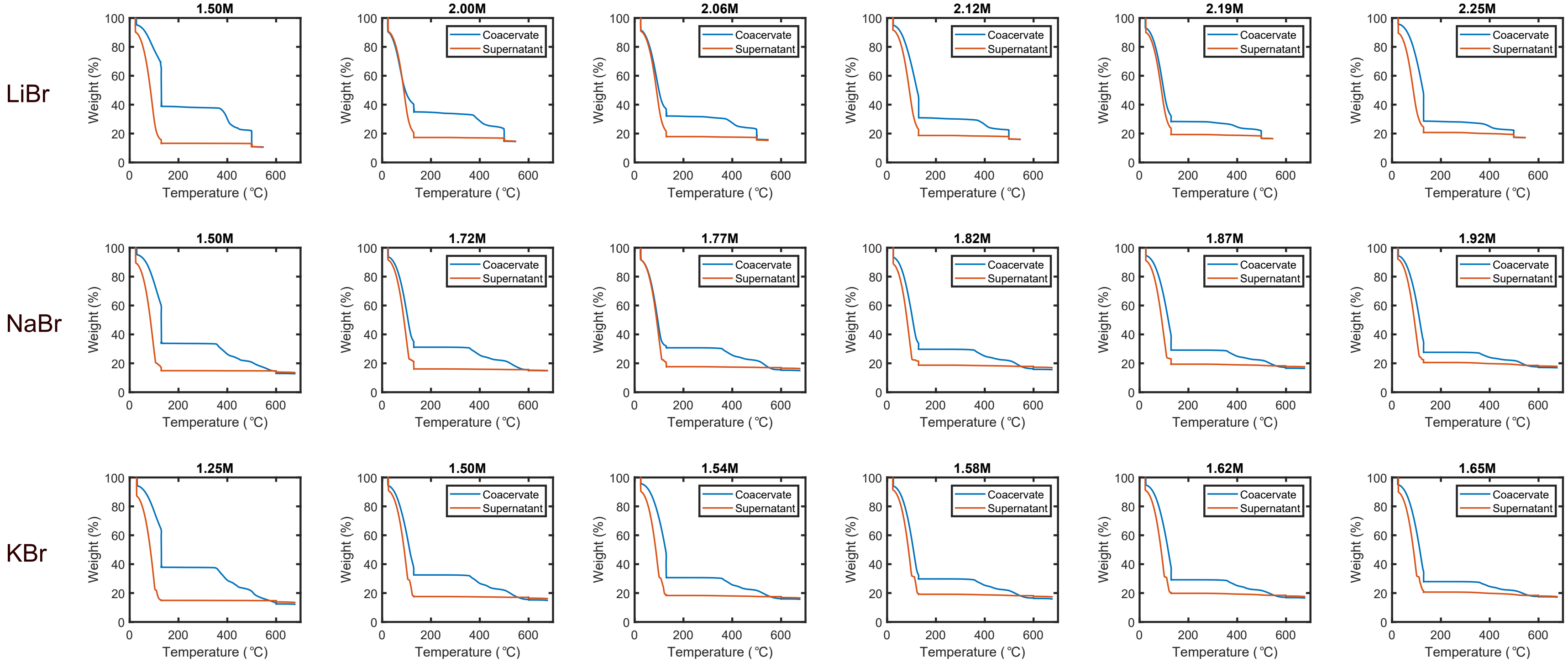

Figure S2: TGA traces for the coacervate and supernatant phases of PSS/PDADMA samples prepared below the binodal.

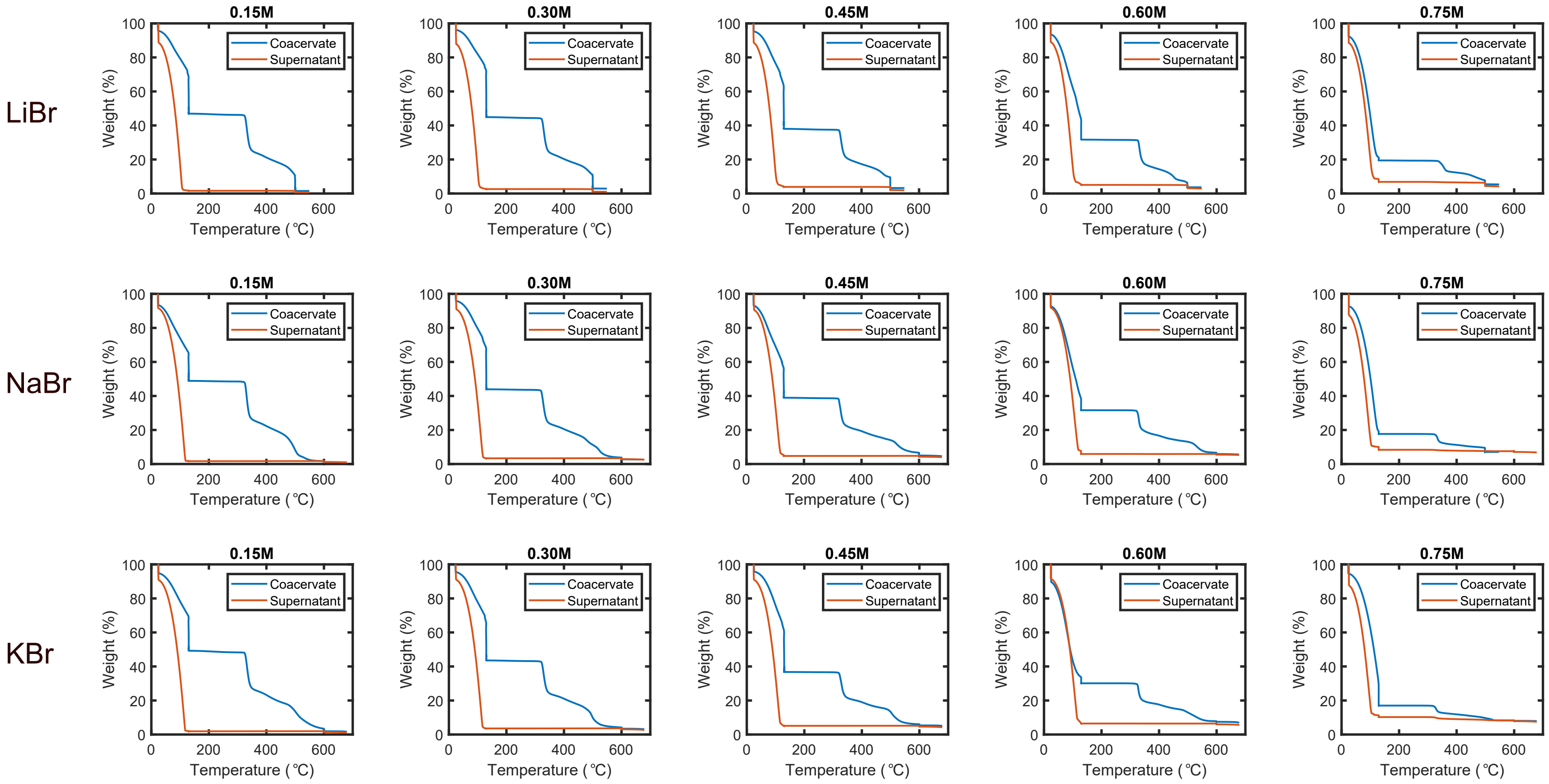

Figure S3: TGA traces for the coacervate and supernatant phases of PAMPS/PDADMA samples prepared below the binodal.

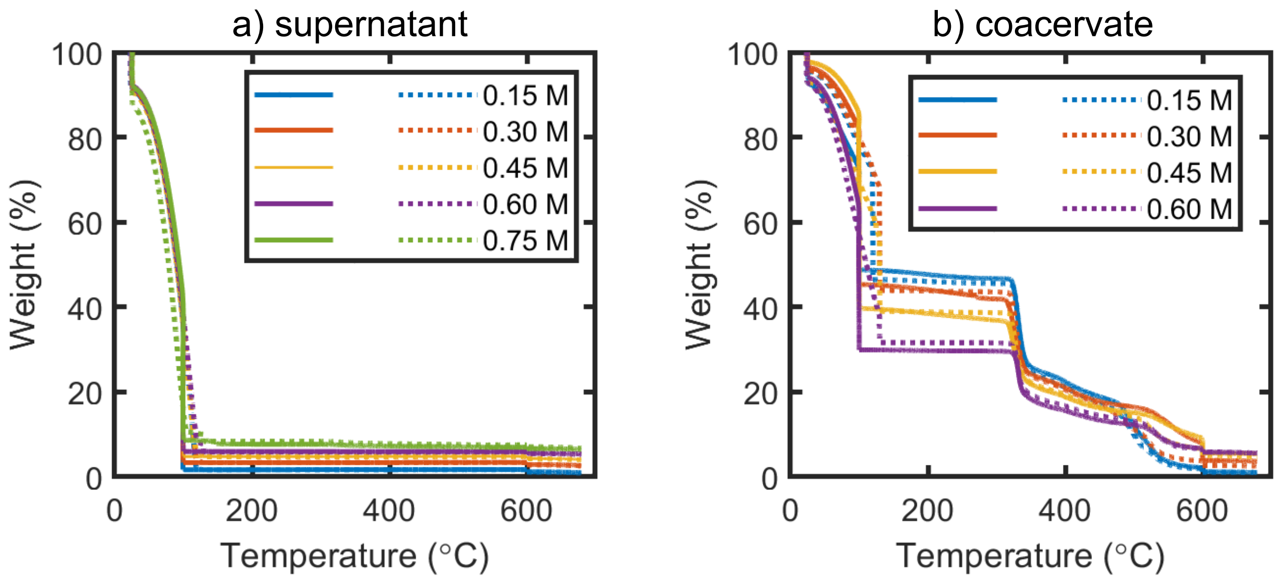

Figure S4: Representative TGA traces from repeat measurements on the (a) supernatant and (b) coacervate phases of samples prepared using NaBr at the salt concentrations given in the plot legends. For each salt concentration, the solid and dotted lines give the TGA traces for two different runs of the same sample.

Compositions of Rheology Samples

The compositions of all samples prepared for rheology measurements are shown in Figs. S5-S6. The binodal compositions determined by TGA are also plotted for reference. As shown in these figures, the compositions of all rheology samples were above the binodal and were expected to form single-phase, homogeneous solutions, as was observed experimentally.

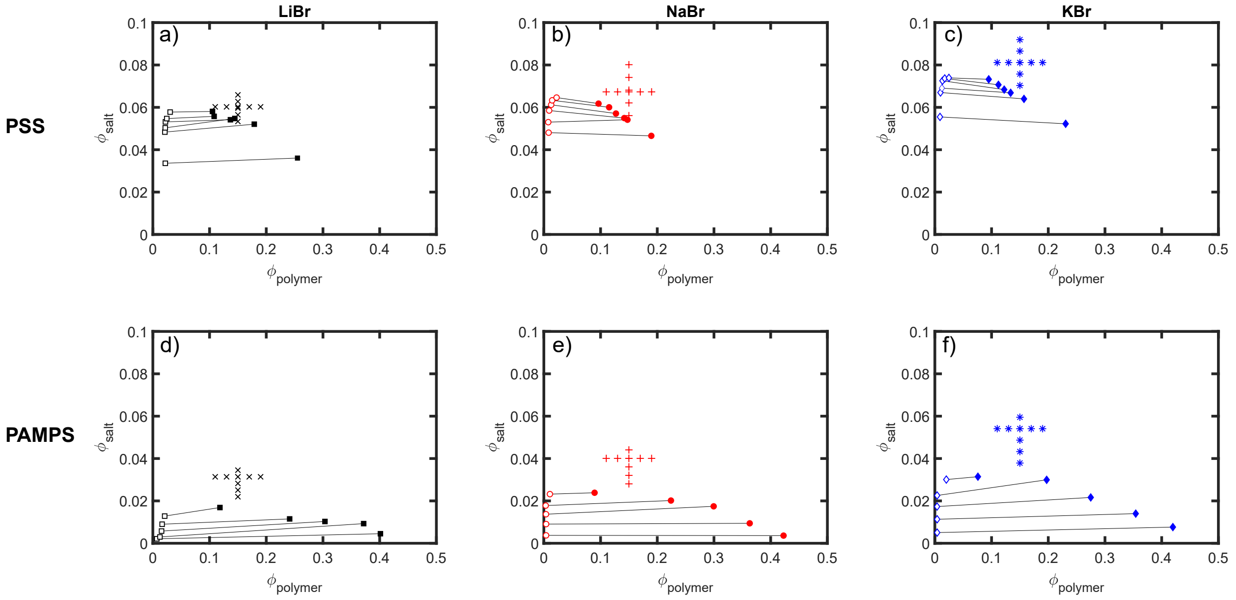

Figure S5: Phase diagrams of (a-c) PSS/PDADMA and (d-f) PAMPS/PDADMA prepared with (a,d) LiBr, (b,e) NaBr, and (c,f) KBr. Open and filled symbols indicate the compositions of the supernatant and coacervate phases, respectively, for samples prepared below the binodal. Symbols above the binodal curve indicate sample compositions used for rheology. Phase diagrams are plotted as a function of the volume fraction of salt and polymer in each sample.

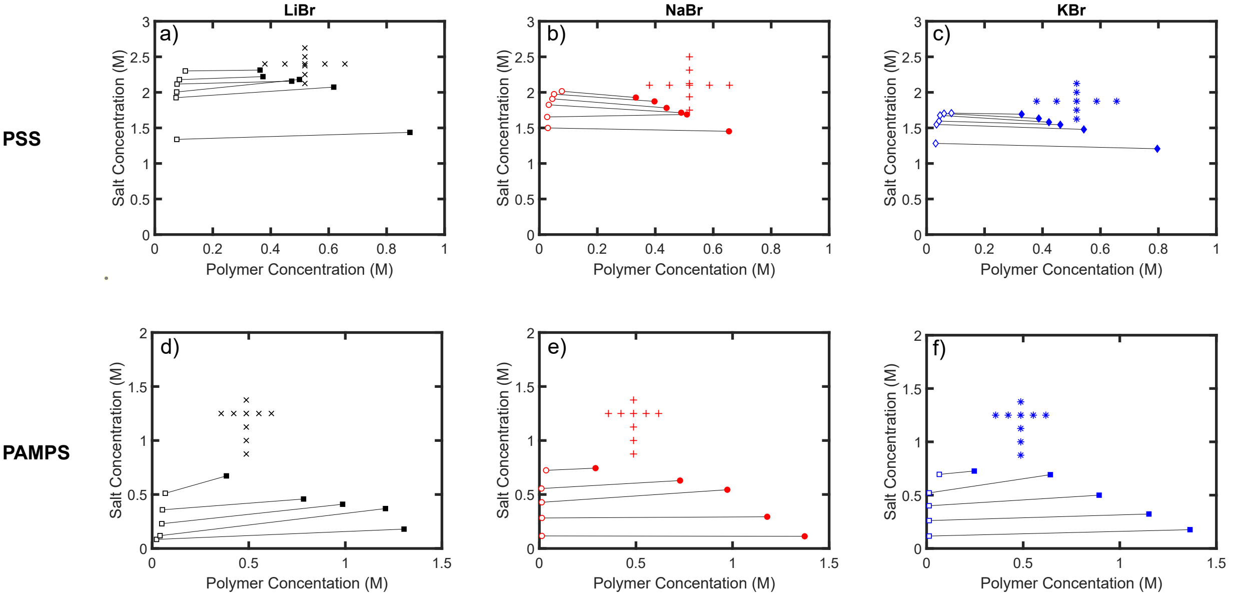

Figure S6: Phase diagrams of (a-c) PSS/PDADMA and (d-f) PAMPS/PDADMA prepared with (a,d) LiBr, (b,e) NaBr, and (c,f) KBr. Open and filled symbols indicate the compositions of the supernatant and coacervate phases, respectively, for samples prepared below the binodal. Symbols above the binodal curve indicate sample compositions used for rheology. Phase diagrams are plotted as a function of the molarity of salt and polymer (repeat unit basis) in each sample.

Rheology

Flow curves, amplitude sweeps, and frequency sweeps for all samples included in the rheology analysis are shown in Figures S7-S12. As noted in the main text, flow curves (Figs. S7-S8) were acquired of shear rates from 0.1-500 s^{-1}. The zero shear viscosities were then acquired by taking the average of the linear portion at low strain rates $\sim$ 0.1-1.0 s^{-1}. Amplitude sweeps (Figs. S9-S10) were conducted at an angular frequency of 10 rad/s over three decades of strain amplitudes, ranging from approximately 0.1% to 100% strain. Frequency sweeps (Figs. S11-S12) were obtained between 600 and 0.1 rad/s; the strain was increased from 0.1% at high frequency to 100% at low frequency to improve sensitivity at low frequencies.[S3] In the frequency sweeps, the strain at 10 rad/s (the frequency at which the amplitude sweeps were conducted) was approximately 2%. As seen in Figs. S9-S10, most samples were in the linear viscoelastic regime at this strain, and most were also linear to strains of 100%. The range of strains giving linear response is expected to increase with decreasing frequency, indicating that the frequency sweeps were in the linear regime. Samples with significant deviations from linearity were generally omitted from further analysis, as discussed in more detail, below.

As with the TGA measurements, repeat flow curves and frequency sweeps were carried out on a subset of the rheology samples to assess the reproducibility in the determined viscosities. Representative traces illustrating the reproducibility of the rheology measurements are shown in Figs. S13 and S14. Across all repeated samples, the standard deviations were typically less than 5% of the average viscosity, yielding error bars smaller than the size of the symbols in Figs. 4 and 7 of the main text.

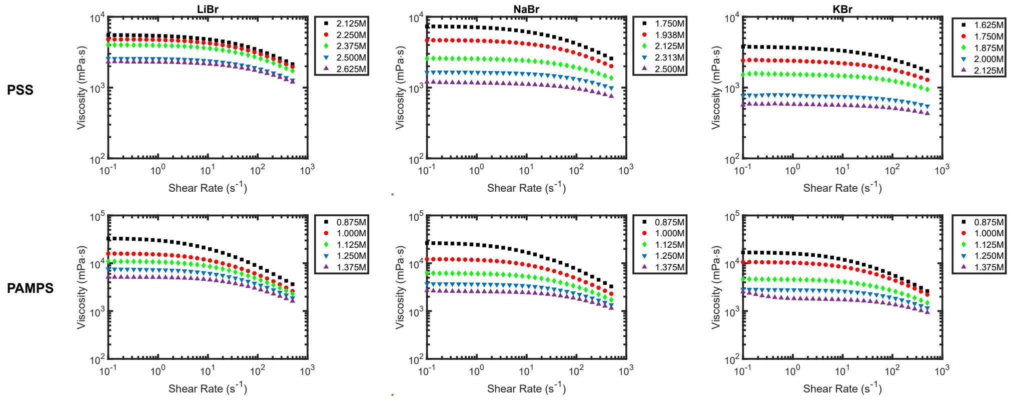

Figure S7: Flow curves of PSS/PDADMA and PAMPS/PDADMA samples prepared with LiBr, NaBr and KBr at constant polymer concentration ($\phi_{pol} = 0.15$) and varying salt concentrations.

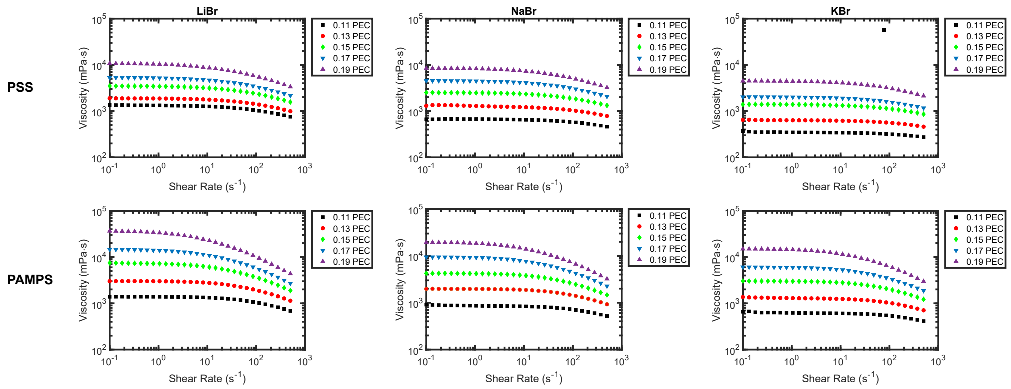

Figure S8: Flow curves of PSS/PDADMA and PAMPS/PDADMA samples prepared with LiBr, NaBr and KBr at different polymer concentrations (legends indicate the volume fraction of polymer in each sample). As discussed in the main text, all PAMPS/PDADMA samples were prepared at a salt concentration of 1.25 M, while the salt concentration for PSS/PDADMA samples varied from 2.4 M for LiBr to 2.1 M for NaBr to 1.875 M for KBr.

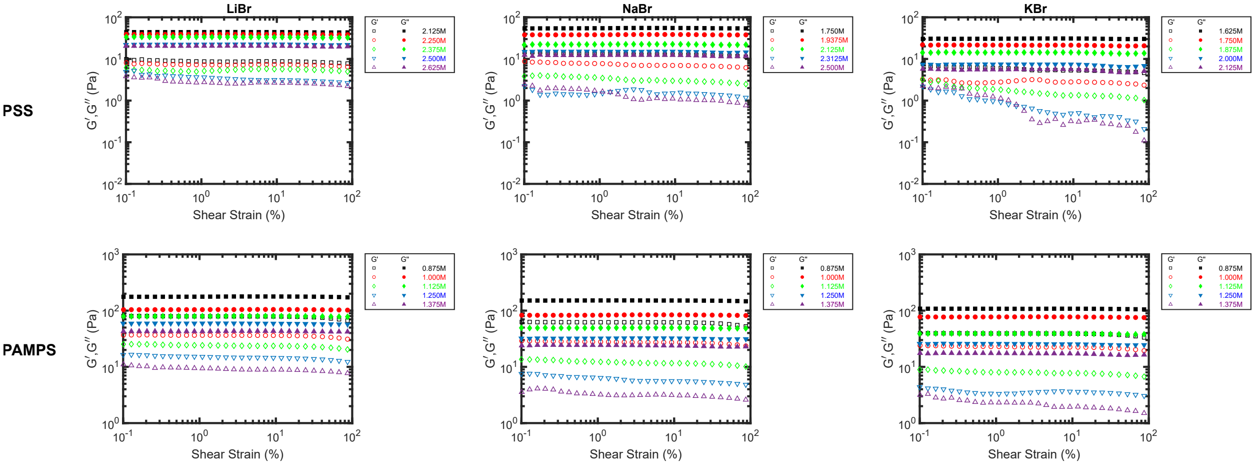

Figure S9: Amplitude sweeps of PSS/PDADMA and PAMPS/PDADMA samples prepared with LiBr, NaBr and KBr at constant polymer concentration ($\phi_{pol} = 0.15$) and varying salt concentrations.

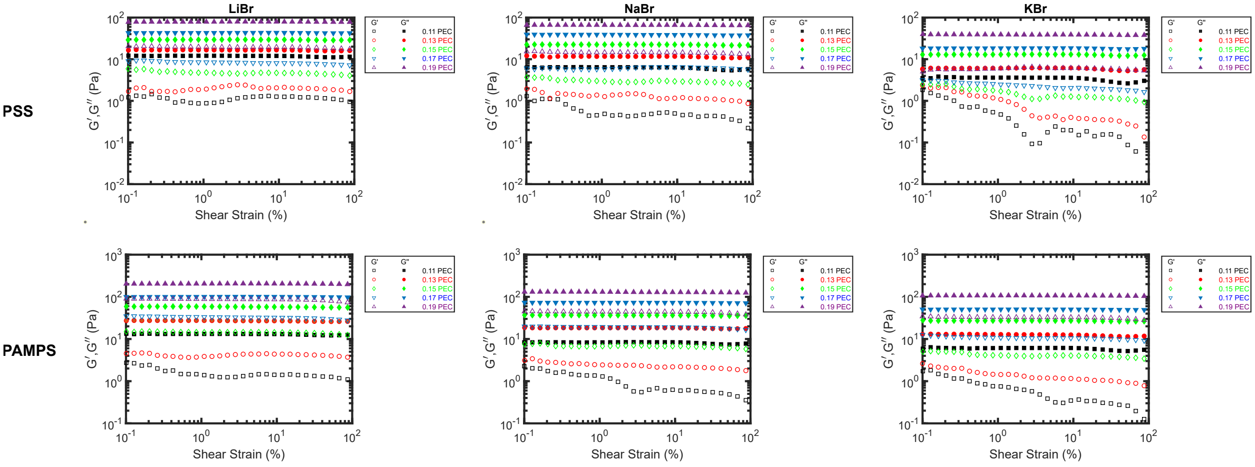

Figure S10: Amplitude sweeps of PSS/PDADMA and PAMPS/PDADMA samples prepared with LiBr, NaBr and KBr at different polymer concentrations (legends indicate the volume fraction of polymer in each sample). As discussed in the main text, all PAMPS/PDADMA samples were prepared at a salt concentration of 1.25 M, while the salt concentration for PSS/PDADMA samples varied from 2.4 M for LiBr to 2.1 M for NaBr to 1.875 M for KBr.

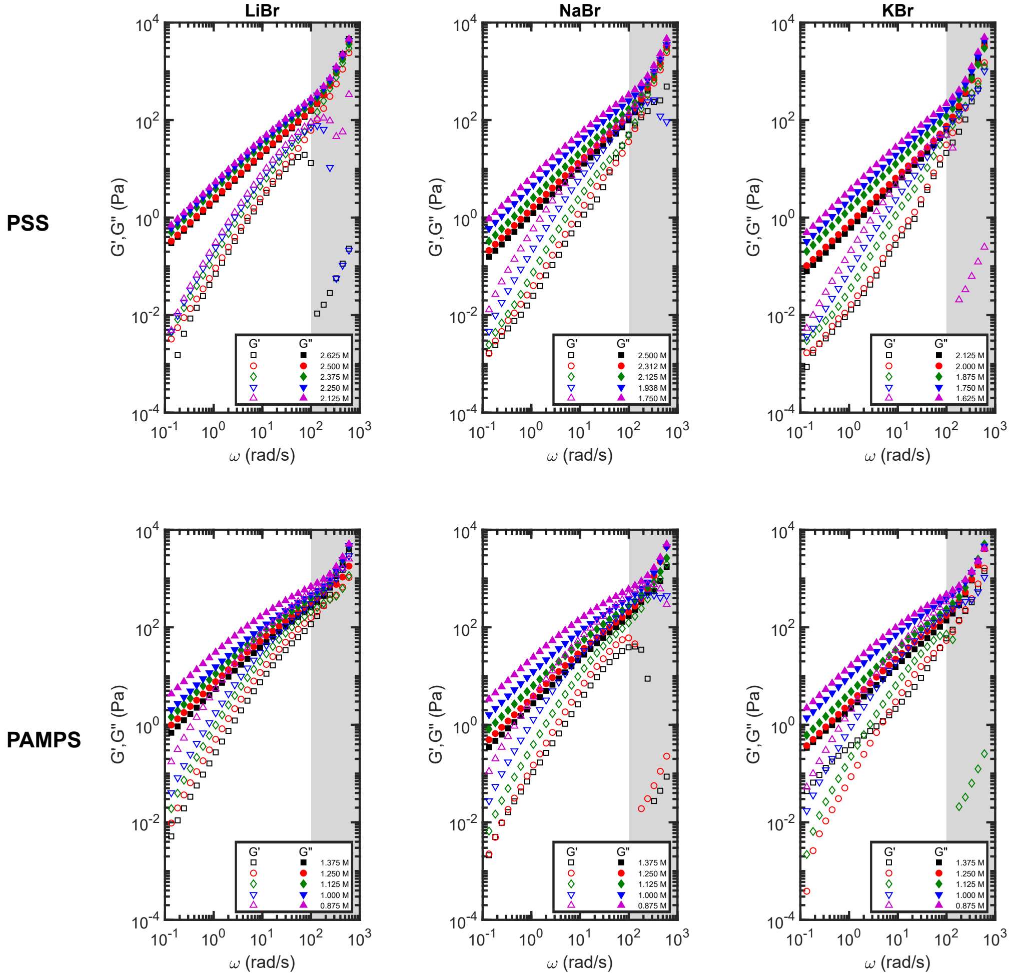

Figure S11: Frequency sweeps of PSS/PDADMA and PAMPS/PDADMA samples prepared with LiBr, NaBr and KBr at constant polymer concentration ($\phi_{pol} = 0.15$) and varying salt concentrations. The shaded grey region indicates data that was unusable due to the inertial limit of the rheometer.

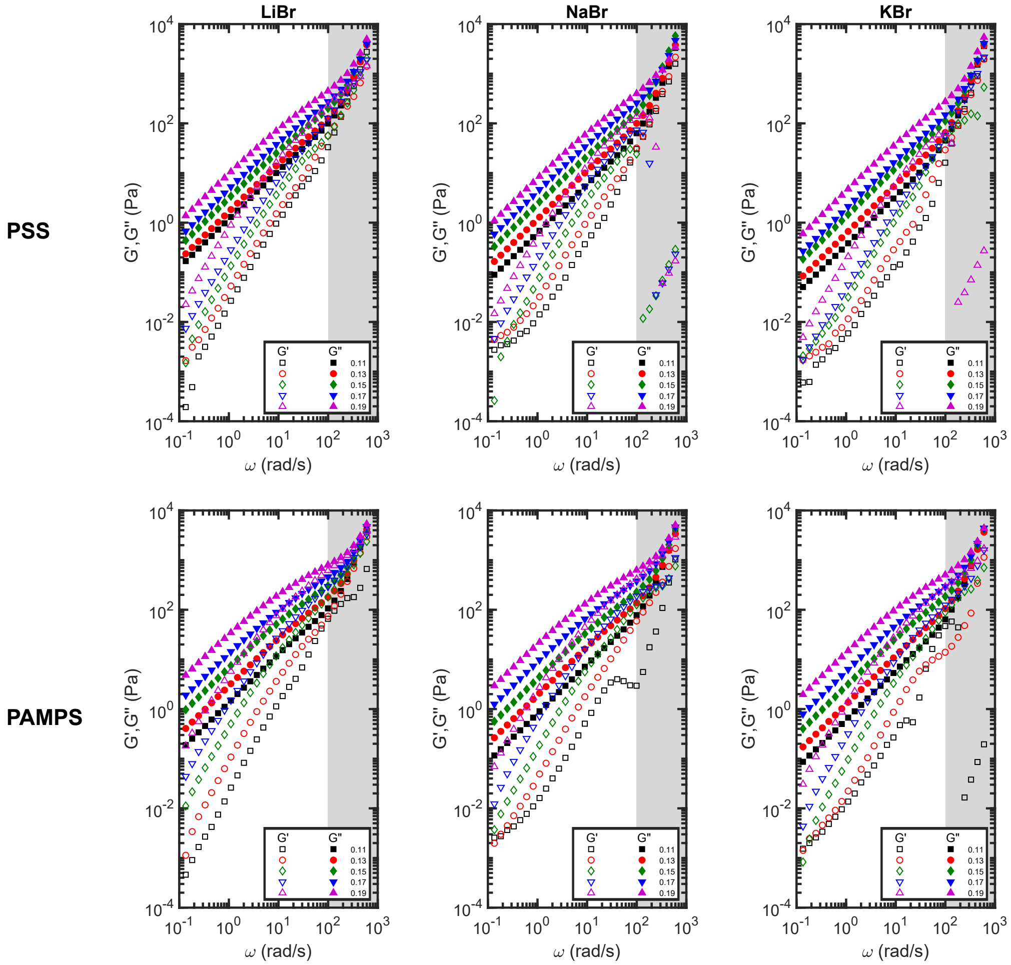

Figure S12: Frequency sweeps of PSS/PDADMA and PAMPS/PDADMA samples prepared with LiBr, NaBr and KBr at different polymer concentrations (legends indicate the volume fraction of polymer in each sample). As discussed in the main text, all PAMPS/PDADMA samples were prepared at a salt concentration of 1.25 M, while the salt concentration for PSS/PDADMA samples varied from 2.4 M for LiBr to 2.1 M for NaBr to 1.875 M for KBr. The shaded grey region indicates data that was unusable due to the inertial limit of the rheometer.

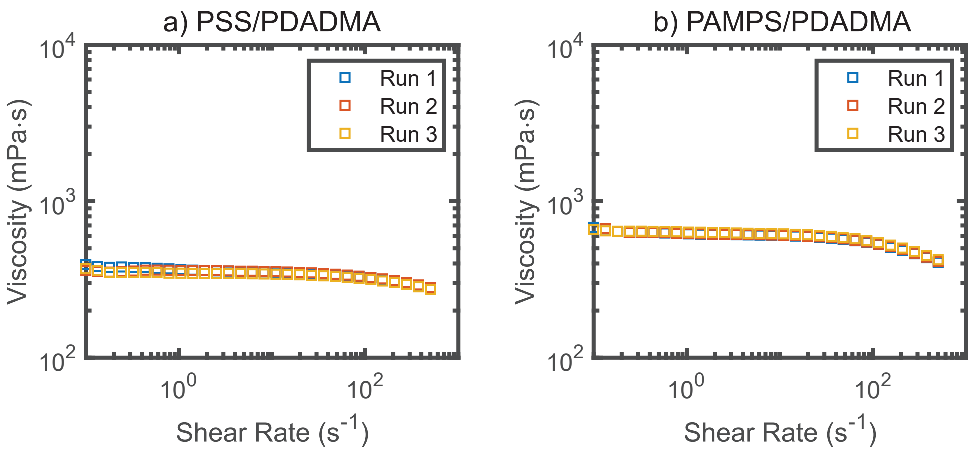

Figure S13: Flow curves measured in repeat measurements on samples of (a) PSS/PDADMA prepared at a volume fraction of polymer of 0.11 and KBr concentration of 1.875 M and (b) PAMPS/PDADMA prepared at a volume fraction of polymer of 0.11 and KBr concentration of 1.25 M.

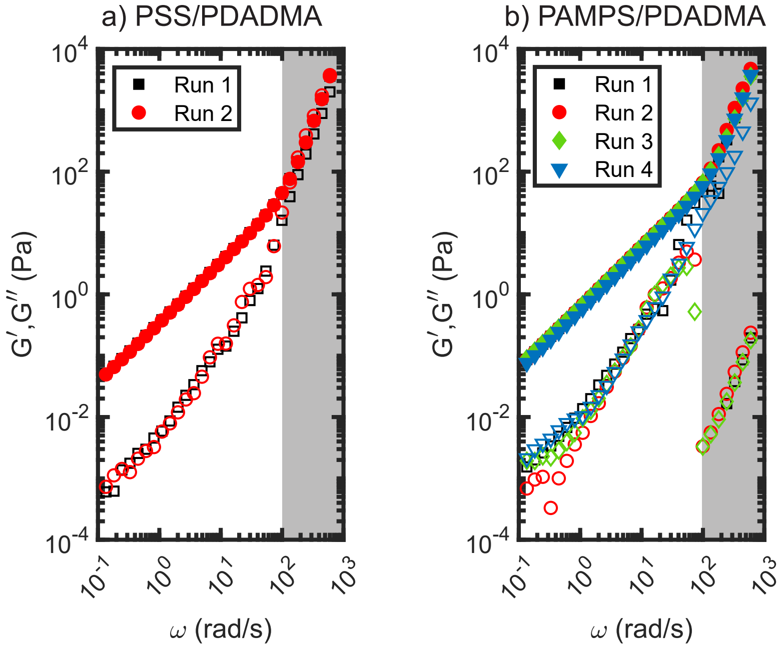

Figure S14: Frequency sweeps measured in repeat measurements on samples of (a) PSS/PDADMA prepared at a volume fraction of polymer of 0.11 and KBr concentration of 1.875 M and (b) PAMPS/PDADMA prepared at a volume fraction of polymer of 0.11 and KBr concentration of 1.25 M. The shaded grey region indicates data that was unusable due to the inertial limit of the rheometer.

Supplemental Analysis

Time-Salt Superposition

As described in the main text, time-salt and time-polymer concentration superpositions were used to determine the horizontal and vertical shift factors (and thus changes in relaxation time and modulus) of the samples. We note that a number of frequency sweeps were omitted from the superposition analysis because they did not exhibit acceptable data quality. In particular, the PAMPS/PDADMA sample prepared with KBr at the highest salt concentration (1.375 M) exhibited significant deviation from terminal scaling at low frequencies. This sample, and a PSS/PDADMA sample prepared at 2.25 M KBr (not shown), were visually observed to become turbid within a few seconds after loading on the rheometer. This suggests that evaporation of water before the evaporation blocker was in place led to phase separation and/or gelation of these samples.[S4] These samples were thus omitted from the superposition analysis. The PSS/PDADMA and PAMPS/PDADMA samples prepared with KBr at a polymer volume fraction of 0.11 also had very low viscosities and noisy amplitude sweeps, and were similarly omitted from the superposition.

The remaining data were cropped to remove instrument response artifacts prior to superposition. Data at high frequencies ($\omega > 10^2$ - see shaded regions in Figs. S11-S12) exhibited signatures of the rheometer's inertial limit, including rapid downturns in G' and/or increases in the scaling of G' and G" to $\sim \omega^2$. For very low viscosity samples, the frequency sweeps also exhibited signatures of low torque errors, including deviation of G' from the expected ω^2 scaling and significant noise in the amplitude sweeps. These limitations restricted the useful frequency range to approximately two decades in the low-viscosity samples; while this narrow frequency range would not be sufficient for true time-salt or time-polymer superposition across the entire relaxation spectrum, it is sufficient for analyzing the relative terminal flow behaviors of the materials.

Modified Cole-Cole plots (Figs. S15-S16) were used to verify that the cropped data

should indeed superpose. Time-salt and time-polymer concentration superpositions were then carried out using the $\tan\delta$ data to obtain the horizontal shift factors and the modulus data to obtain the vertical shift factors. The superposed $\tan\delta$ plots are shown in Figs. S17-S18, and the corresponding master curves are shown in Figs. S19-S20. As shown in these figures, the data did indeed superpose satisfactorily across the entire salt and polymer concentration series.

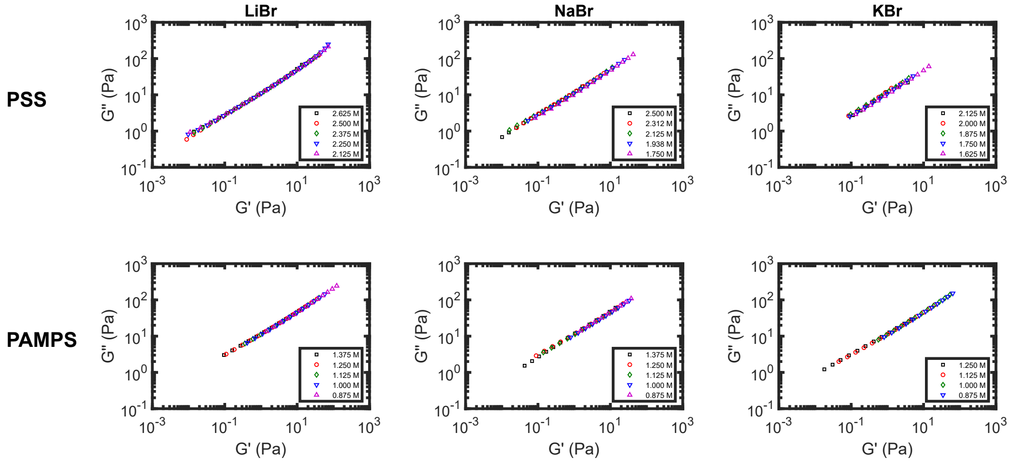

Figure S15: Modified Cole-Cole plots of PSS/PDADMA and PAMPS/PDADMA samples prepared at constant polymer concentration ($\phi_{pol} = 0.15$) and varying salt concentrations. Data was cropped to remove instrument artifacts, as described in the text.

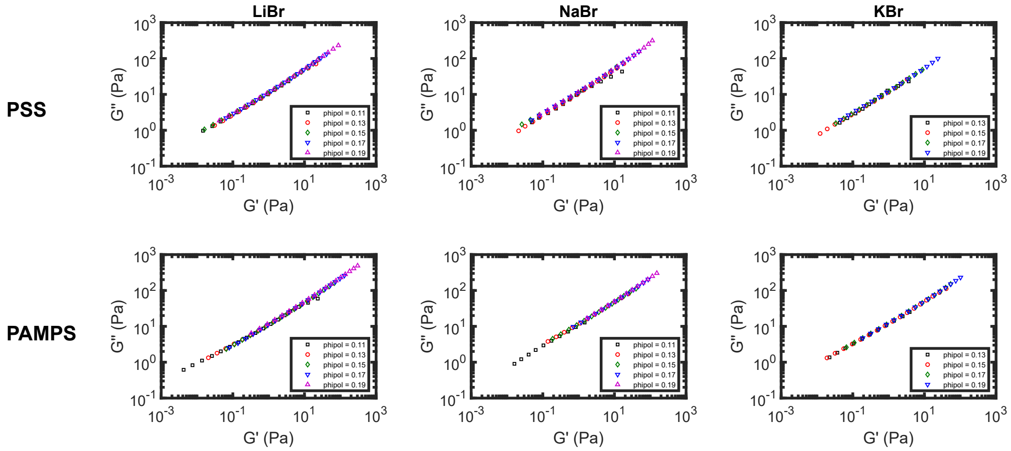

Figure S16: Modified Cole-Cole plots of PSS/PDADMA and PAMPS/PDADMA samples prepared at varying polymer concentrations. As discussed in the main text, all PAMPS/PDADMA samples were prepared at a salt concentration of 1.25 M, while the salt concentration for PSS/PDADMA samples varied from 2.4 M for LiBr to 2.1 M for NaBr to 1.875 M for KBr. Data was cropped to remove instrument artifacts, as described in the text.

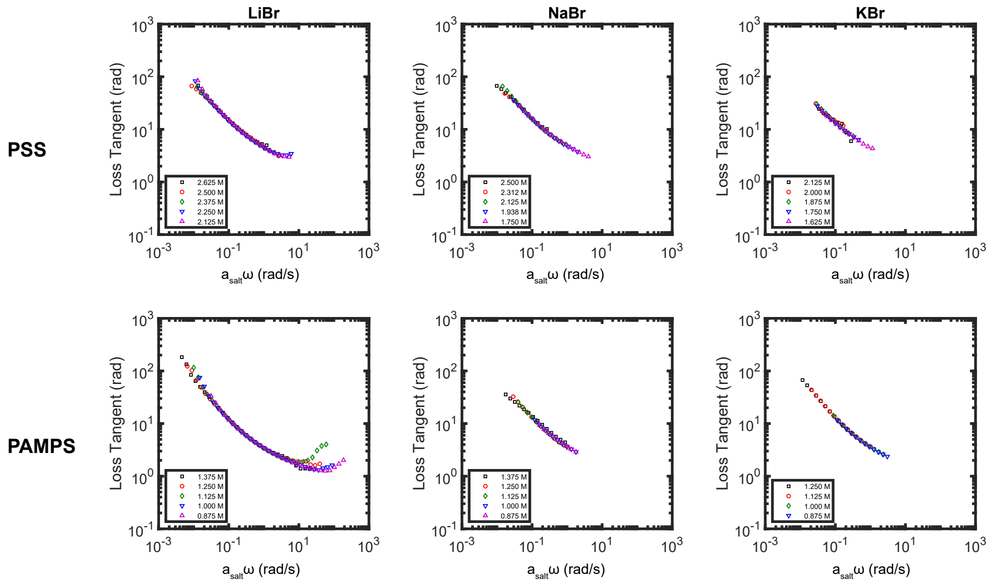

Figure S17: Shifted $\tan\delta$ plots of PSS/PDADMA and PAMPS/PDADMA samples prepared at constant polymer concentration ($\phi_{pol} = 0.15$) and varying salt concentrations. Data was cropped to remove instrument artifacts, as described in the text.

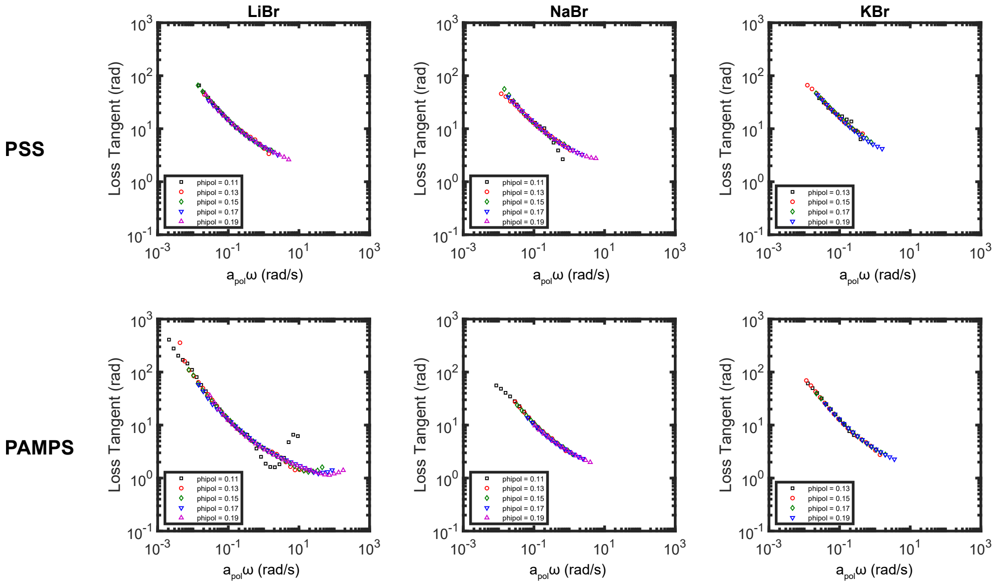

Figure S18: Shifted $\tan\delta$ plots of PSS/PDADMA and PAMPS/PDADMA samples prepared at varying polymer concentrations. As discussed in the main text, all PAMPS/PDADMA samples were prepared at a salt concentration of 1.25 M, while the salt concentration for PSS/PDADMA samples varied from 2.4 M for LiBr to 2.1 M for NaBr to 1.875 M for KBr. Data was cropped to remove instrument artifacts, as described in the text.

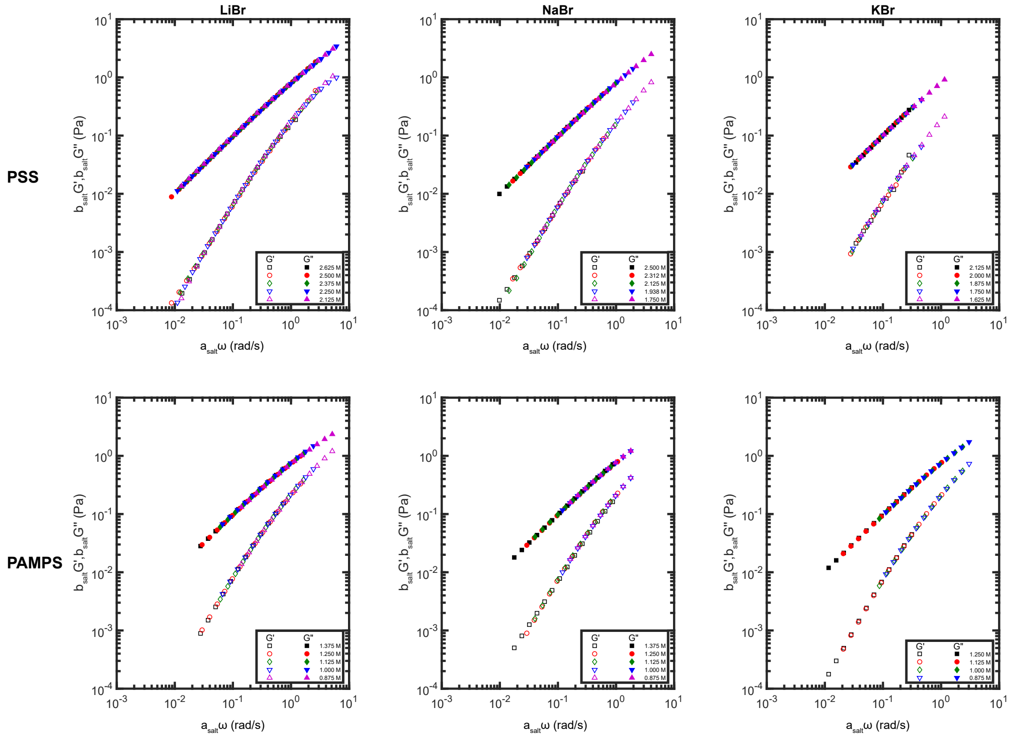

Figure S19: Master Curves of PSS/PDADMA and PAMPS/PDADMA prepared with LiBr, NaBr, and KBr at constant polymer concentration ($\phi_{pol} = 0.15$) and varying salt concentrations. Data was cropped to remove instrument artifacts, as described in the text.

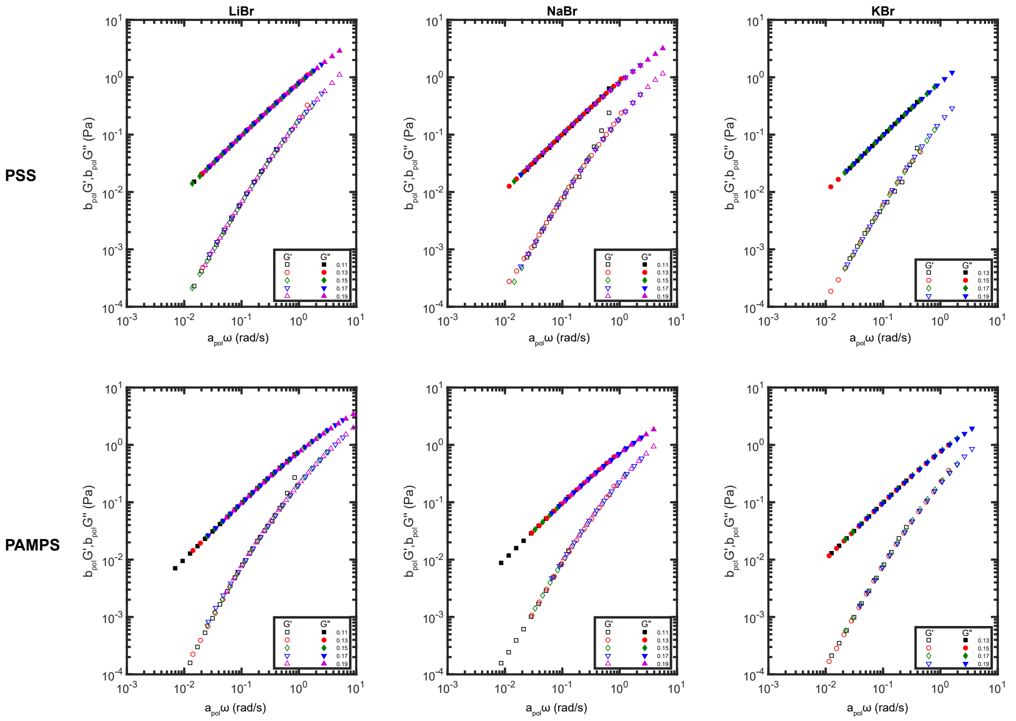

Figure S20: Master Curves of PSS/PDADMA and PAMPS/PDADMA prepared with LiBr, NaBr, and KBr at varying polymer concentrations. As discussed in the main text, all PAMPS/PDADMA samples were prepared at a salt concentration of 1.25 M, while the salt concentration for PSS/PDADMA samples varied from 2.4 M for LiBr to 2.1 M for NaBr to 1.875 M for KBr. Data was cropped to remove instrument artifacts, as described in the text.

Fits to Determine Crossover Point

The frequencies and moduli at the crossover point (and thus changes in relaxation time and modulus) were also estimated from the intersection of linear fits to the low frequency portions of G' and G''. A narrow range of G' and G'' values (at frequencies of approximately 1-10 rad/s) were fit to $G \sim \omega^{\alpha}$. Fits to G'' yielded $\alpha \approx 0.97 \pm 0.03$, while fits to G' yielded $\alpha \approx 1.6 \pm 0.1$, suggesting that the data in the fitted range was close to the terminal regime. The crossover point was determined from the intersection of the fits to G' and G'', and τ was determined from the inverse of ω at the crossover point. The resulting values of τ and G are shown in Figs. S21-S22. As shown in these figures, the trends for both τ and G for both the salt and polymer series yielded similar trends to those obtained using the superposition analysis described above.

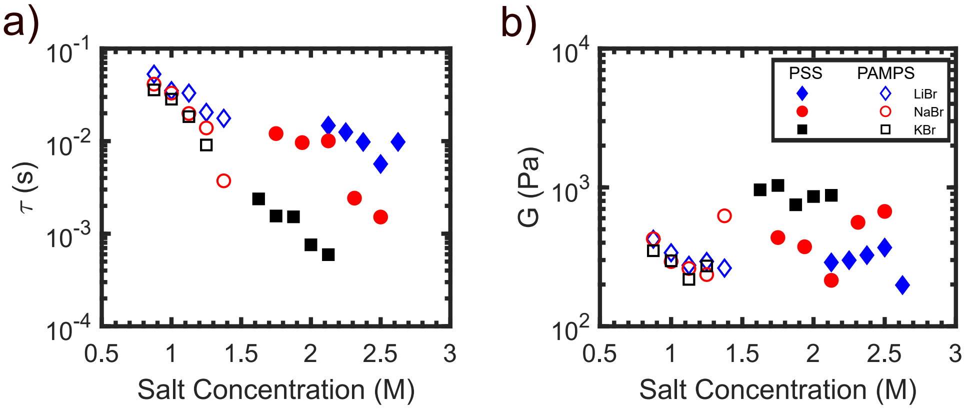

Figure S21: Relaxation time, τ and crossover moduli, G, for PSS/PDADMA and PAMPS/PDADMA samples prepared with LiBr (blue diamonds), NaBr (red circles), and KBr (black squares) at constant polymer concentration ($\phi_{pol} = 0.15$) and varying salt concentrations.

Polymer Scaling

As noted in the main text, relaxation times and viscosities for materials following "sticky" Rouse dynamics are expected to scale as[S5,S6]

$$\tau \sim e^{E_a(c_{salt})} \phi_{pol}^{\beta} \tag{3}$$

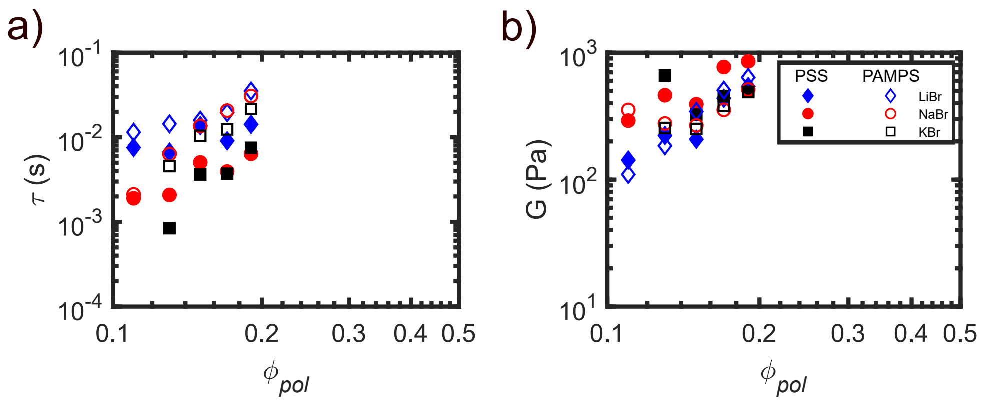

Figure S22: Relaxation time, τ and crossover moduli, G, for PSS/PDADMA and PAMPS/PDADMA samples prepared with LiBr (blue diamonds), NaBr (red circles), and KBr (black squares) at varying polymer concentrations. As discussed in the main text, all PAMPS/PDADMA samples were prepared at a salt concentration of 1.25 M, while the salt concentration for PSS/PDADMA samples varied from 2.4 M for LiBr to 2.1 M for NaBr to 1.875 M for KBr.

and

$$\eta \sim \phi_{pol}\tau \sim e^{E_a(c_{salt})}\phi_{pol}^{\beta+1} \tag{4}$$

where E_a is the salt-dependent activation energy for sticker rearrangement and β is a scaling exponent describing the dependence on polymer concentration.[S6,S7] When salt concentration is held constant,

$$\tau \sim \phi_{pol}^{\beta} \tag{5}$$

and

$$\eta \sim \phi_{pol}^{\beta+1} \tag{6}$$

Scaling exponents extracted from fits of the viscosities and shift factors for the data sets in which salt concentration was held constant are summarized in Table S1. As seen in this table, fits to the viscosities yielded $\beta = 2.8 - 3.6$ and $\beta = 4.8 - 5.0$ for PSS/PDADMA and PAMPS/PDADMA, consistent with the values predicted for the non-renormalized ($\beta = 3.2$) and renormalized ($\beta = 5.0$) sticky Rouse model, respectively.[S5] Scaling exponents extracted from the horizontal shift factors were slightly higher ($\beta = 3.5 - 4.2$ for PSS/PDADMA and $\beta = 5.2 - 5.9$ for PAMPS/PDADMA), while fits of the vertical shift factors, which

are proportional to the inverse of the modulus, yielded scaling exponents of -0.3 to -0.8, or slightly weaker than the $G^{-1} \sim \phi_{pol}^{-1}$ scaling expected for un-entangled polyelectrolyte solutions.[S8]

Table S1: Parameters for fits of samples with constant salt concentration to $\eta \sim \phi_{pol}^{\beta_\eta+1}$, $a_{pol} \sim \phi_{pol}^{\beta_a}$, and $b_{pol} \sim \phi_{pol}^{\beta_b}$

PEC	Salt	β_η	β_a	β_b
	LiBr	2.8 ± 0.6	3.5 ± 0.6	-0.5 ± 0.1
PSS	NaBr	3.6 ± 0.2	4.0 ± 0.4	-0.8 ± 0.2
	KBr	3.5 ± 0.5	4.2 ± 0.8	-0.8 ± 0.2
	LiBr	5.0 ± 0.5	5.5 ± 0.3	-0.5 ± 0.3
PAMPS	NaBr	5.0 ± 0.5	5.2 ± 0.5	-0.8 ± 0.1
	KBr	4.8 ± 0.5	5.9 ± 1.1	-0.3 ± 0.1

In the complex coacervate literature, the salt-dependent activation energy is typically assumed to obey $\frac{E_a}{kT} = -A\sqrt{c_{salt}} + B$.[S6,S9] While this expression is only strictly valid in dilute salt solutions and is likely not rigorously applicable to the high salt concentration samples used here,[S9] fits to the form $\tau \sim e^{-A\sqrt{c_{salt}}}$ and $\eta \sim e^{-A\sqrt{c_{salt}}}$ were also carried out for the data sets in which polymer concentration was held constant. These fits, summarized in Table S2, yielded values of A between 6 and 12, consistent with values in the range expected for salt solutions with concentrations between 1-2 M.[S4]

Table S2: Parameters for fits of samples with constant polymer concentration to $\eta \sim e^{-A_\eta\sqrt{c_{salt}}}$ and $a_{salt} \sim e^{-A_a\sqrt{c_{salt}}}$.

PEC	Salt	A_η	A_a
	LiBr	5.9 ± 1.0	5.7 ± 0.7
PSS	NaBr	7.3 ± 0.4	8.4 ± 1.2
	KBr	10.7 ± 0.9	11.7 ± 1.9
	LiBr	7.5 ± 0.8	7.8 ± 0.8
PAMPS	NaBr	9.8 ± 1.0	10.0 ± 1.2
	KBr	9.8 ± 0.8	11.1 ± 1.0

References

(S1) Shamoun, R. F.; Hariri, H. H.; Ghostine, R. A.; Schlenoff, J. B. Thermal Transformations in Extruded Saloplastic Polyelectrolyte Complexes. *Macromolecules* **2012**, *45*, 9759–9767, DOI: 10.1021/ma302075p.

(S2) Chee, C. H.; Benharush, R.; Knight, L. R.; Laaser, J. E. Segregative Phase Separation of Strong Polyelectrolyte Complexes at High Salt and High Polymer Concentrations. *Soft Matter* **2024**, DOI: 10.1039/D4SM00994K.

(S3) Velankar, S. S.; Giles, D. How do I know my phase angles are correct? *Rheol. Bull.* **2007**, *76*, 8–20.

(S4) Morin, F. J.; Puppo, M. L.; Laaser, J. E. Decoupling salt- and polymer-dependent dynamics in polyelectrolyte complex coacervates via salt addition. *Soft Matter* **2021**, *17*, 1223–1231, DOI: 10.1039/d0sm01412e.

(S5) Rubinstein, M.; Semenov, A. N. Thermoreversible Gelation in Solutions of Associating Polymers. 2. Linear Dynamics. *Macromolecules* **1998**, *31*, 1386–1397, DOI: 10.1021/ma970617+.

(S6) Spruijt, E.; Cohen Stuart, M. A.; van der Gucht, J. Linear Viscoelasticity of Polyelectrolyte Complex Coacervates. *Macromolecules* **2013**, *46*, 1633–1641, DOI: 10.1021/ma301730n.

(S7) Liu, Y.; Winter, H. H.; Perry, S. L. Linear viscoelasticity of complex coacervates. *Advances in Colloid and Interface Science* **2017**, *239*, 46–60, DOI: 10.1016/j.cis.2016.08.010.

(S8) Colby, R. H. Structure and linear viscoelasticity of flexible polymer solutions: comparison of polyelectrolyte and neutral polymer solutions. *Rheologica Acta* **2009**, *49*, 425–442, DOI: 10.1007/s00397-009-0413-5.

(S9) Larson, R. G.; Liu, Y.; Li, H. Linear viscoelasticity and time-temperature-salt and other superpositions in polyelectrolyte coacervates. *Journal of Rheology* **2021**, *65*, 77–102, DOI: 10.1122/8.0000156.
